# NSUN2 mediated-aberrant 5-methylcytosine methylation regulates autophagy-related ferroptosis in oral squamous cell carcinoma progression

**DOI:** 10.1038/s41419-025-08174-y

**Published:** 2025-12-23

**Authors:** Yunyang Lu, Runze Li, Weidong Du, Jie Wu, Yi He, Lingyu Yuan, Xun Chen, Shiyu Lv, Fangyang Shi, Jiajun Hu, Wei Zhao, Dongsheng Yu

**Affiliations:** 1 Hospital of Stomatology, Guanghua School of Stomatology, Guangdong Provincial Key Laboratory of Stomatology, Sun Yat-sen University, Guangzhou, Guangdong, China; 2 Department of Oral and Maxillofacial Surgery, Shandong Provincial Hospital Affiliated to Shandong First Medical University, Jinan, Shandong China

**Keywords:** Oral cancer, Macroautophagy, Cancer therapeutic resistance

## Abstract

Oral squamous cell carcinoma (OSCC) is a common malignant tumor with high metastasis rates and poor prognosis. This study investigated the role of NOP2/Sun RNA methyltransferase family member 2 (NSUN2), a key 5-methylcytosine (m5C) methyltransferase, and m5C methylation in the progression of OSCC, particularly in relation to ferroptosis resistance. NSUN2 is significantly overexpressed in OSCC tissues and cell lines and its high expression correlates with poor prognosis and aggressive tumor characteristics. Knockdown of NSUN2 in ferroptosis-resistant OSCC cells resulted in increased sensitivity to ferroptosis. Conversely, NSUN2 overexpression conferred ferroptosis resistance, reducing iron accumulation and restoring GPX4 expression even under erastin treatment. Mechanistically, NSUN2 mediates m⁵C modification of sequestosome 1 (SQSTM1)/P62 mRNA, and the m5C reader protein Y-box binding protein 1 (YBX1) enhances SQSTM1/P62 mRNA stability. This regulation suppresses autophagy and thereby inhibits autophagy-dependent ferroptosis in OSCC. In vivo xenograft models confirmed that NSUN2 knockdown significantly inhibited tumorigenicity. Notably, treatment with an autophagy inhibitor (3-MA) or a ferroptosis inhibitor (Fer-1) partially restored tumor growth in NSUN2-knockdown cells, validating the critical role of autophagy and ferroptosis in NSUN2-mediated OSCC progression. These findings identify the NSUN2-YBX1-SQSTM1/P62 axis as a key regulator of autophagy-dependent ferroptosis in OSCC, highlighting NSUN2 as a promising epitranscriptomic target to enhance ferroptosis induction for OSCC therapy.

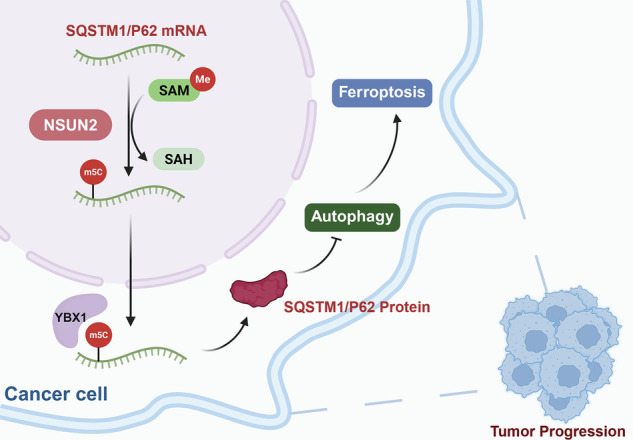

## Introduction

Oral squamous cell carcinoma (OSCC) represents the most prevalent form of head and neck malignancy and is attributed mainly to tobacco and/or alcohol consumption as well as betel nut chewing [1]. Despite the application of surgical resection combined with chemoradiotherapy for many years, the high recurrence rate and frequent occurrence of lymph node metastasis have led to the overall 5-year survival rate of OSCC patients remaining below 50% in recent years, with no significant progress in the past two decades [2]. In addition to the removal of pathological factors, it is crucial to focus on developing new treatments to effectively reduce OSCC mortality. Although research has shown that modulating apoptosis [3], pyroptosis [4], necrosis [5], and other cell death mechanisms can produce partial antitumor effect in OSCC, a number of issues remain unresolved. Our prior work disclosed that the ferroptosis-related gene were considered vital targets for treating tumors and predicting prognosis [6] and that alterations in N6-methyladenosine (m6A) methylation can modulate ferroptosis, impacting the radiosensitivity of oral squamous cell carcinoma [7]. Moreover, numerous studies have verified the effectiveness of ferroptosis as an anti-cancer strategy [8–10]. However, the resistance of OSCC cells to ferroptosis can lead to drug resistance and significantly reduce the effectiveness [11–13]. Thus, the aim of this study is to elucidate the epigenetic pathways associated with ferroptosis in OSCC.

Ferroptosis is a form of cell death that depends on iron and lipid peroxidation and is characterized by increased iron storage and lipid peroxidation, which cause membrane damage and cell death [14]. This process involves several signaling pathways, such as the rat sarcoma signaling pathway, p53 signaling pathway, Hippo signaling pathway, hypoxia inducible factor 1, alpha signaling pathway, etc. [15–17]. Moreover, increasing evidence suggests that autophagy is strongly linked to ferroptosis, indicating that ferroptosis relies on autophagy [18, 19]. Studies have indicated that autophagy-dependent ferroptosis can modulate drug resistance, the tumor immune microenvironment, metabolic reprogramming and nanomedicine therapy in cancer [19]. Ferroptosis can be controlled through various molecular mechanisms and processes, including epigenetic regulation, transcriptional or posttranscriptional regulation, and mitochondrial dysfunction [19–22]. Therefore, our study focused primarily on the posttranscriptional regulation of autophagy-dependent ferroptosis, providing a more comprehensive understanding of its therapeutic potential.

RNA 5-methylcytosine (m5C) is a frequent posttranscriptional modification. This process dynamically depends on the activities of methylases, demethylases and m5C reader proteins [23]. This modification is crucial for RNA metabolic processes, including processing, export, translation and stability [19, 24–26]. Modification of m5C can be catalyzed by the Nol1/Nop2/SUN (NSUN) domain family [27]. The α-ketoglutarate-dependent dioxygenase ABH1 (ALKBH1) and ten-eleven translocation family proteins (TETs) can remove m5C modifications on tRNAs and mRNAs, respectively [28]. The Aly/REF export factor (ALYREF) and y-box binding protein 1 (YBX1) can recognize and bind the m5C motif in mRNA and then mediate export or affect the stability of the mRNA [29, 30]. An in-depth investigation of the interaction between m5C modification levels and ferroptosis in OSCC can provide more effective therapeutic strategies for the treatment of OSCC [31].

In the present study, we investigated the expression status of NOP2/Sun RNA methyltransferase family member 2 (NSUN2) and the functional role of m5C methylation in OSCC, specifically its impact on ferroptosis sensitivity through autophagy modulation. We hypothesize that increased NSUN2 activity not only enhances m5C modification of specific mRNAs, promoting OSCC cell survival and resistance to ferroptosis but also regulates autophagy in a way that further influences ferroptosis pathways. Our findings revealed that NSUN2 interacts with YBX1, a recognized m5C reader protein, to stabilize target mRNAs critical for OSCC cell proliferation, metastasis, and autophagy-related processes. By elucidating the NSUN2-YBX1 pathway, this research aimed to identify novel therapeutic targets that could help overcome ferroptosis resistance through autophagy regulation, ultimately improving treatment outcomes for OSCC patients.

## Materials and methods

### Pathological samples and database analysis of TCGA data

OSCC specimens and adjacent noncancerous tissues (ANCTs) were obtained from the Hospital of Stomatology, Sun Yat-Sen University. Patients who had received chemotherapy or radiation therapy prior to surgery were not included. Prior to the pathological test and biopsy, written informed consent was obtained. This study complies with the Declaration of Helsinki and was authorized by the hospital’s ethical committee (approval number KQEC-2024-34-01).

Publicly available RNA-seq data from the TCGA TARGET GTEx pan-cancer cohort (PANCAN, N = 19,131 samples) were obtained via the UCSC Xena Browser (https://xenabrowser.net). Normalized gene expression values, measured in transcripts per million (TPM), were extracted for NSUN2. Expression levels were log-transformed [log₂(TPM + 1)] and visualized across tumor and normal tissues for 34 distinct cancer types using the ggplot2 R package (v3.3.6). The OSCC cohort was curated based on the following inclusion criteria: overall survival exceeding 30 days and primary tumor location restricted to the alveolar ridge, buccal mucosa, floor of mouth, hard palate, oral cavity, or oral tongue. Overall survival (OS) data for 298 TCGA-OSCC patients (filtered for OS > 30 days) were analyzed. The survminer R package (v0.4.9) surv_cutpoint function was used to determine the optimal cutpoint for NSUN2 expression data. The survival R package (v3.4.0) survfit function was utilized to create survival curves, which were then plotted using the survminer R package ggsurvplot function. Univariate and multivariate Cox proportional hazards regression analyses were performed using the survival R package coxph function. The ggplot2 package was used to visualize hazard ratios (HR) and 95% confidence intervals (CI).

Univariate and multivariate Cox proportional hazards models (survival R package) assessed associations between NSUN2 expression (continuous variable), age, gender, TNM stage, and OS. HR and 95% CI were reported. Differentially expressed genes (DEGs) between NSUN2-high and NSUN2-low groups were identified using DESeq2 ( | log₂FC | ≥ 1, padj < 0.05). A counts expression matrix (distinct from normalized data) was used as input for DESeq2. The results of differential gene expression analysis were visualized using the ggplot2 R package. GO enrichment analysis was performed using the clusterProfiler package (v4.2.2) enrichGO function, with a P-value < 0.05 set as the threshold for statistical significance.

### Single-cell RNA sequencing (scRNA-seq) data processing

Gene Expression Omnibus (GEO) dataset GSE172577 is a single-cell transcriptomic dataset in oral cancer. The dimensionality reduction and clustering of the dataset were performed using Seurat (v4.3.0). Cells expressing >200 genes and with <20% mitochondrial reads were retained. Cell annotation was conducted by comparing the differentially expressed genes of each cell cluster with marker genes of known cell types. t-Distributed Stochastic Neighbor Embedding (t-SNE) was used to visualize the annotated cell populations. The scCancer package (https://github.com/wguo-research/scCancer) was employed to distinguish normal epithelial cells from tumor cells. The annotated data were then used to analyze the expression and distribution of m5C regulators, ferroptosis drivers, and suppressors. Specifically, m5C regulators included NSUN2, NSUN3, NSUN4, NSUN5, NSUN6, NSUN7, ALKBH1, ALYREF, YBX1, TET1, TET2, and TET3; ferroptosis drivers and suppressors were retrieved from FerrDb (http://www.zhounan.org/ferrdb/current/).

### Hematoxylin and eosin, IHC and IF staining

An H&E staining kit (Solarbio, G1120-100) was used for staining. A 3% H2O2 solution was used for antigen retrieval, and the samples were blocked via immunohistochemistry. The sections were then incubated with primary anti-Ki67 (Abcam, ab16667; 1:500) or anti-NSUN2 (Proteintech, 20854-1-AP; 1:200) or anti-sequestosome 1 (SQSTM1) /P62 (Abcam, ab109012; 1:500) overnight at 4 °C. The secondary antibody was then administered. After that, hematoxylin was applied as a counterstain, and DAB was used for chromogenic detection. The percentage of cells that were positively stained (0–100) and the staining intensity (0–3) were used to calculate the IHC score.

For IF staining, after incubation with the primary antibody at 4 °C overnight, the slides were treated with the secondary antibody (Cy3 goat anti-rabbit IgG (H + L), ABclonal, AS007; 1:200 dilution) for 1 h at room temperature in 3% BSA/PBS after being incubated with the primary antibody overnight at 4 °C. An Olympus fluorescence microscope was used to view and take pictures of the samples after they had been blocked with a blocking agent that included DAPI.

### Cell Culture and Transfection

ScienCell (Carlsbad, CA, USA) provided the NOK cells. We acquired CAL27, HSC6, SCC15, HSC3, SAS, UM1, and HEK293T cells from the American Type Culture Collection. Dulbecco’s modified Eagle’s medium (Gibco, C11995500BT) with a high glucose content and 10% fetal bovine serum was used to cultivate the cells. Every cell line was incubated in a humidified environment with 5% CO_2_ at 37 °C.

We purchased lentiviruses LV-Control, LV-shNSUN2 and LV-OE NSUN2 from Genechem Biotechnology in Shanghai, China. To produce the lentivirus, the lentiviral vectors were cotransfected into HEK293T cells along with the packaging vectors psPAX2 and pMD2.G. Puromycin was used to select stable gene-expressing cell lines.

Additionally, the negative control (siCtrl) and YBX1 siRNA were acquired from GeneChem Co. (Shanghai, China). Cell transfection was performed via Lipofectamine 3000 (Invitrogen, L3000008).

The sequences of siRNAs and shRNAs were shown in Table S3.

### Western blot analysis

Western blotting was carried out in accordance with our earlier procedure [73]. NSUN2 (Affinity, DF12103; 1:1000), SQSTM1/P62 (Abcam, ab109012; 1:500), YBX1 (Abcam, ab76149; 1:1000), ACSL4 (Affinity, DF12141; 1:1000), GPX4 (Affinity, DF6701; 1:1000), SLC11A2 (Affinity, DF12740; 1:1000), Transferrin (Cell Signaling Technology, 35293; 1:1000), BECN1 (Cell Signaling Technology, 3495; 1:1000), LC3A/B (Cell Signaling Technology, 4108; 1:1000), ATG5 (Cell Signaling Technology, 12994; 1:1000) and β-actin (Affinity, AF7018; 1:1000) were among the primary antibodies used to incubate the membranes. Anti-mouse secondary antibody (Cell Signaling Technology, 7076; 1:2000) or anti-rabbit secondary antibody (Cell Signaling Technology, 7074; 1:2000) was then incubated with the membranes.

### RNA Extraction and qRT-PCR

qRT-PCR was carried out in accordance with our earlier methodology [32]. The relative mRNA levels were calculated using β-actin as the endogenous control to normalize the data. The primer sequences for qRT-PCR are presented in Table S2.

### Cell proliferation, colony formation, migration and invasion assays

In accordance with our previous protocol, cell proliferation, colony formation, migration and invasion assays were performed [73].

### Lipid peroxidation assay

A six-well plate was used to seed cells prior to treatment. The cells were treated with 5 μM C11BODIPY (Thermo Fisher Scientific, #D3861) in HBSS at 37 °C for 30 min after the culture media was removed. The cells were digested with trypsin and then centrifuged after being washed twice with PBS. For flow cytometric analysis, the cells were resuspended in serum-free medium, and FlowJo software was used to analyze the data.

### TEM

TEM was performed according to our previously described protocol [74]. A Philips CM10 transmission electron microscope was used to capture images.

### Measurement of Fe^2+^ levels

A FerroOrange fluorescent probe (Dojindo, F374) was used to measure the levels of Fe^2+^. A working solution of 1 μmol/L FerroOrange probe was then applied to the cells in accordance with the manufacturer’s instructions. The cells were incubated for 30 min at 37 °C in the dark. Finally, a fluorescence microscope was used to view the cells and take pictures. ImageJ software was used to quantify the fluorescence intensity.

### Dot Blot Assay and Global m5C Measurement

Following denaturation at 90 °C, total RNA was spotted onto a nylon membrane (Solarbio, YA1760) and crosslinked with UV radiation (120 mJ/cm^2^). The membranes were blocked with anti-m5C (Proteintech, 68301-1-lg; 1:5000), anti-m6A (Proteintech, 68055-1-Ig; 1:5000) or anti-m7G (Proteintech, 68302-1-Ig; 1:5000) antibodies and then incubated overnight at 4 °C. The secondary antibody was then added. Finally, chemiluminescence was employed for detection, with 0.02% methylene blue staining serving as a control.

An m5C RNA Methylation Quantification Kit (Epigentek, P-9009-96) was used to colorimetrically quantify the amount of m5C in total RNA to assess m5C globally. Two hundred nanograms of RNA was applied to each detection well, and the m5C level was then measured. The calculations were performed via a conventional curve.

### m5C-Bis-Seq, mRNA-Seq, and bioinformatics analysis

E-GENE Tech Co., Ltd. (Shenzhen, China) performed m5C-Bis-Seq and mRNA-Seq in accordance with the manufacturer’s instructions. Reliable sites were defined as having a methylated cytosine depth of at least 2, a coverage depth of at least 10, and a m5C methylation level of at least 0.1. Finally, the distribution of m5C sites was visualized via the metaPlotR program (https://github.com/olarerin/metaPlotR).

For every experimental condition, three separate biological replicates were produced for mRNA-Seq. The identification of differentially expressed mRNAs was based on a log2-fold change ( ≥ 1) and an adjusted p value (<0.05).

### Molecular docking and molecular dynamics simulations

The potential for NSUN2 protein to form a stable complex with SQSTM1/P62 mRNA was evaluated via molecular docking experiments. The three-dimensional structure of the protein was predicted using AlphaFold, followed by structural optimization through force field construction. The optimized structure was then imported into Gauss09 software for energy minimization. On the HDock platform, the target protein was used as both receptor and ligand for docking and scoring analysis. PyMOL software was employed to visualize the geometric configuration of the binding site, with docking affinity parameters annotated in the three-dimensional model.

### RIP

HSC6 cells were seeded in 10 cm dishes and cultured for 12 hfollowed by transfection with either wild-type/mutant SQSTM1/P62 plasmids or shCtrl/shNSUN2 constructs. After 48 hours of transfection, cells were harvested and processed using a commercial RNA immunoprecipitation kit (BersinBio, Bes5101S). For m5C-RIP assays following plasmid transfection, total RNA was isolated with TRIzol reagent and incubated overnight at 4 °C with Protein A/G Dynabeads conjugated to either anti-m⁵C antibody (ab10805, Abcam) or mouse IgG control (ab131368, Abcam). For experiments involving shRNA transfection, cells were lysed in RIP lysis buffer on ice for 30 min, and the lysates were incubated with beads coated with anti-m5C, anti-YBX1, or corresponding IgG controls for 4 h at room temperature. The complexes were washed five times with ice-cold washing buffer, and bound RNA was recovered using TRIzol. qRT-PCR was performed on both input RNA (1% of total) and immunoprecipitated samples. Relative enrichment of m5C-modified or protein-bound RNA was calculated using the 2⁻ΔCt method, normalizing to the input control. The plasmids of WT- SQSTM1/P62 and MUT- SQSTM1/P62 were constructed by inserting the wild-type sequence or the mutant sequence into the pcDNA3.1 plasmid, respectively.

### RNA stability assay

Actinomycin D (5 μg/mL) treatment of SCC9/HSC6 cells was used to determine the RNA stability in NSUN2-stably knocked down cells. Total RNA was then extracted for qRT‒PCR at predetermined intervals of 0, 1, 2, and 3 h. The amount of time needed for a 50% decrease in mRNA expression was determined to be the half-life (t1/2).

### Detection of ROS levels

A DCFH-DA fluorescent probe (Beyotime, S0033) was used to measure the levels of ROS in the cells. After cell transfection, HSC6 and SCC9 cells were plated in 12-well plates at a density of 5 × 10^4^ cells per well, as directed by the manufacturer. The cells were then treated with erastin (10 μM) or not for 24 h. Following three washes with PBS, the cells were treated with 10 μM DCFH-DA for 20 min at 37 °C in the dark. Finally, the cells were examined under a fluorescence microscope. ImageJ software was used to measure the intracellular ROS levels.

### GSH:GSSG ratio assay

GSH and GSSG were quantified with GSH and GSSG Assay Kits (Beyotime, S0053).

### Double-labeled lentivirus mRFP-GFP-LC3 transfection

HSC6 and SCC9 cells were seeded into confocal Petri dishes and maintained in culture for 24 h Subsequently, the cells were transfected with mRFP-GFP-LC3 lentivirus (Hanbio, Shanghai, China) following the manufacturer’s instructions.

### Xenograft tumor models

In accordance with the 3 R principles, male BALB/c nude mice aged 5–6 weeks were randomly allocated to one of four groups: Control, shNSUN2, Con + shNSUN2, shNSUN2 + Autophagy Inhibitor (3-MA), or shNSUN2 + Ferroptosis Inhibitor (Fer-1). Each group had eight biological replicates. Under anesthesia, a subcutaneous injection of approximately 1 × 10^7^ HSC6 cells was performed into the left axilla. Every three days, the body weights of the mice were measured. To obtain subcutaneous xenografts, the animals were sacrificed by cervical dislocation while under pentobarbital sodium anesthesia. The embedded tissues were sectioned and processed for histological analysis. Prior to the pathological test and biopsy, written informed consent was obtained. This study complies with the Declaration of Helsinki. The Institutional Animal Care and Use Committee of SYSU granted approval for all experimental and animal care procedures (approval number SYSU-lACUC-2023-001535).

### Statistical analysis

The results are shown as the means ± SDs of biological replicates unless otherwise noted. Two-tailed Student’s t tests or chi-square tests for tumor grade were used to determine the significance of differences in unpaired parameters between two groups. Log-rank tests and Kaplan‒Meier curves were used for survival analysis. The level of statistical significance was established at a threshold of p < 0.05. GraphPad Prism 9.5 (GraphPad Software, San Diego, CA, USA) and SPSS 27 (IBM SPSS, Armonk, NY, USA) were used for data management and statistical analysis.

## Results

### Elevated NSUN2 expression in OSCC tissues and cells is correlated with poor prognosis

**Fig. 1:**
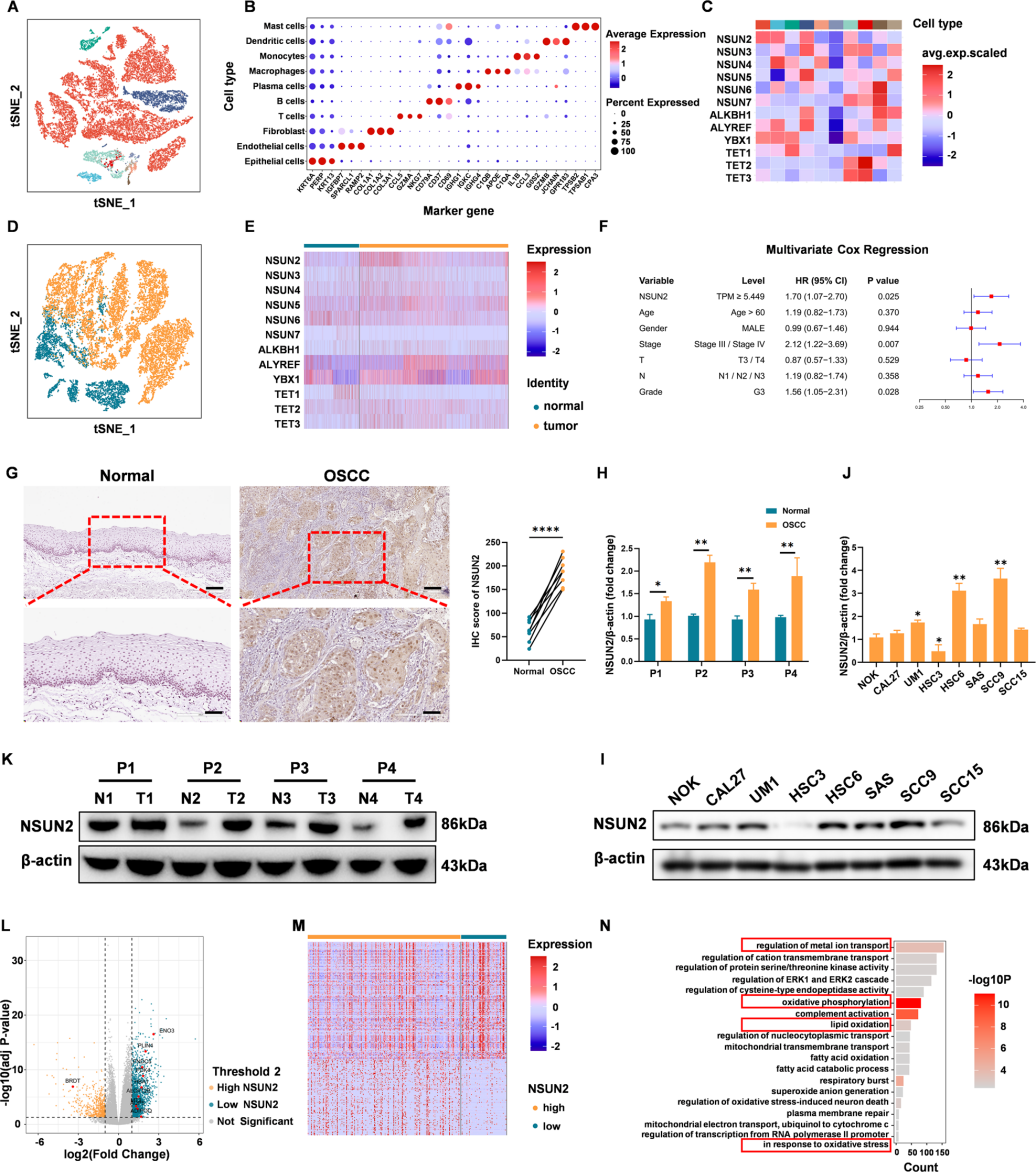
NSUN2 overexpression associated with transcriptomic remodeling and poor prognosis in OSCC. **A** t-SNE projection of single-cell RNA sequencing (scRNA-seq) data (GSE172577) showing immune cell clusters in HNSCC tissues. **B** Dot plots displaying marker genes for distinct cell populations. **C** Heatmap of m5C-related gene expression across OSCC cell types (color bar indicates cell type). **D** t-SNE plot of epithelial cell subpopulations annotated using the scCancer R package. **E** Heatmap showing differential expression of m5C-related genes between malignant and normal epithelial cells. **F** Multivariate Cox regression analyses of overall survival and clinicopathological features in OSCC patients. **G** NSUN2 IHC scores and representative IHC images of OSCC tumor and normal tissues. **H**, **I** Western blot analysis showing the NSUN2 expression tendencies of four OSCC patients. **J**, **K** NSUN2 protein expression were measured using western blotting in NOK, CAL27, UM1, HSC3, HSC6, SAS, SCC9, and SCC15 cells, respectively. **L** Volcano plot depicting DEGs between NSUN2-high and NSUN2-low groups in HNSC. **M** Heatmap of all differentially expressed genes across subtypes (yellow: high expression; blue: low expression). **N** GO enrichment analysis of up- and down-regulated DEGs in HNSC. *P < 0.05, **P < 0.01, and ****P < 0.0001.

First, we examined the pattern of NSUN2 expression in various malignancies. The expression status of NSUN2 was examined via a TNM plot across a range of cancer types in The Cancer Genome Atlas (TCGA). Figure S1A demonstrated that the expression level of NSUN2 was greater in a number of tumor tissues than in neighboring normal tissues, including head and neck squamous cell carcinoma (HNSC). This finding was validated specifically in OSCC, where NSUN2 mRNA levels were significantly elevated in tumor tissues versus matched normal controls (Figure S1B). Patients with high NSUN2 expression had a considerably worse prognosis than those with low NSUN2 expression (Figure S1C). Single-cell RNA sequencing of HNSCC tissues (GSE172577) first resolved this relationship within tumor microenvironments. t-SNE projection identified 12 distinct cellular clusters (Fig. 1A), with marker gene confirmation of epithelial, immune, and stromal populations (Fig. 1B). Critically, malignant epithelial cells exhibited striking enrichment of NSUN2 (log2FC = 4.1, adj.P < 1e-15) and its co-regulator YBX1 (Fig. 1C). Subclustering further confirmed NSUN2 dominance in malignant epithelial subpopulations (inferCNV>0.1; Fig. 1D), which showed widespread dysregulation of m5C-related genes versus normal epithelia (Fig. 1E). Univariate (Fig. S1D) and multivariate Cox regression (Fig. 1F) confirmed NSUN2 as an independent prognostic factor after adjusting for age, gender, and TNM stage. NSUN2 was markedly elevated in tumor tissues compared with nearby control tissues (Fig. 1G). The western blotting results further revealed that NSUN2 expression levels were significantly higher in tumor samples than normal samples (Fig. 1H, I). Among OSCC cell lines, NSUN2 levels were elevated in UM1, HSC6, and SCC9 compared to NOK cells, downregulated in HSC3, and unchanged in other lines (Fig. 1J, K). Transcriptomic profiling of NSUN2-high versus -low groups identified 2,524 differentially expressed genes (Fig. 1L, M), with GO enrichment highlighting regulation of pathways associated with ferroptosis such as metal ion transport, oxidative phosphorylation, lipid oxidation, and in response to oxidative stress, offering directions for future investigations. Critically, baseline clinicopathological analysis (Table S1) demonstrated balanced distribution between NSUN2-high and -low groups. In summary, our findings suggest that NSUN2 is significantly differentially expressed in OSCC and could be an essential factor in the development of cancer.

### Knockdown of NSUN2 suppressed the proliferation, migration and invasion of OSCC cells

**Fig. 2:**
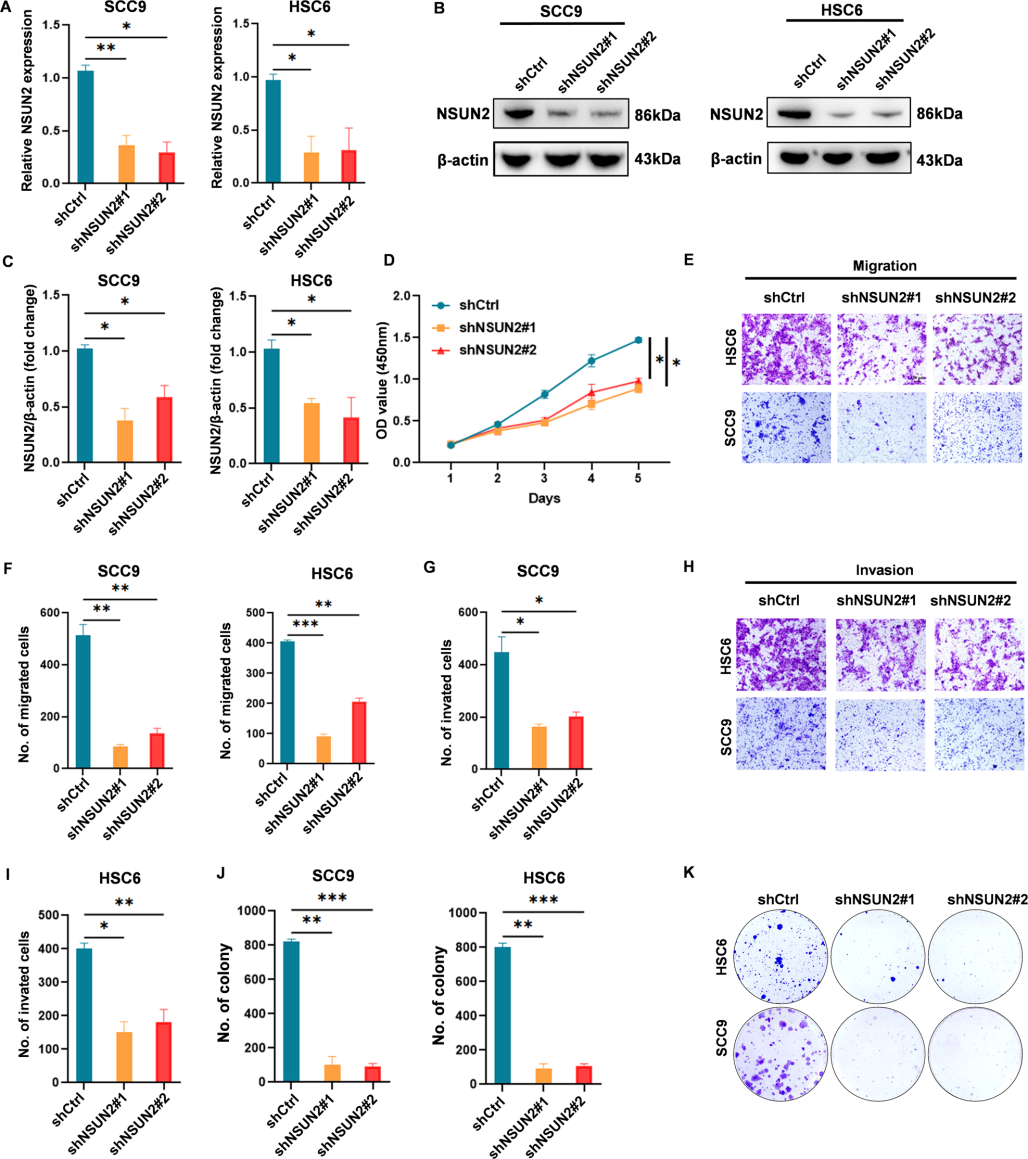
In vitro, OSCC cell invasion, migration, and proliferation are all inhibited by NSUN2 knockdown. NSUN2 mRNA expression (**A**) andNSUN2 protein expression (**B and C**) in SCC9 and HSC6 cells with NSUN2 knockdown by shRNAs #1 and #2. **D** In cells with stable NSUN2 knockdown, cell viability was assessed using the CCK8 test. **E–I** Transwell assays were used to assess the migration and invasion capabilities of SCC9 and HSC6 cells with NSUN2 silencing. **J**, **K** Assays for colony development. *P < 0.05, **P < 0.01, and ***P < 0.001.

To determine the biological function of NSUN2 in NSUN2-high expressed OSCC cells, we successfully transfected these cells with lentiviral vectors containing NSUN2 short hairpin RNA. This resulted in a knockdown efficiency of over 70%, which was verified by qRT-PCR (Fig. 2A) and western blot analysis (Fig. 2B, C). Notably, the knockdown of NSUN2 expression led to a significant reduction in the proliferation of ferroptosis-resistant OSCC cells (Fig. 2D). Additionally, stable NSUN2 knockdown suppressed cell migration and invasion (Fig. 2E–I) and reduced the colony formation ability of resistant cells (Fig. 2J, K). We used shNSUN2#1 in subsequent studies because of its more consistent knockdown effectiveness.

### NSUN2 knockdown enhances ferroptosis sensitivity in oscc cells, while NSUN2 overexpression confers ferroptosis resistance

**Fig. 3:**
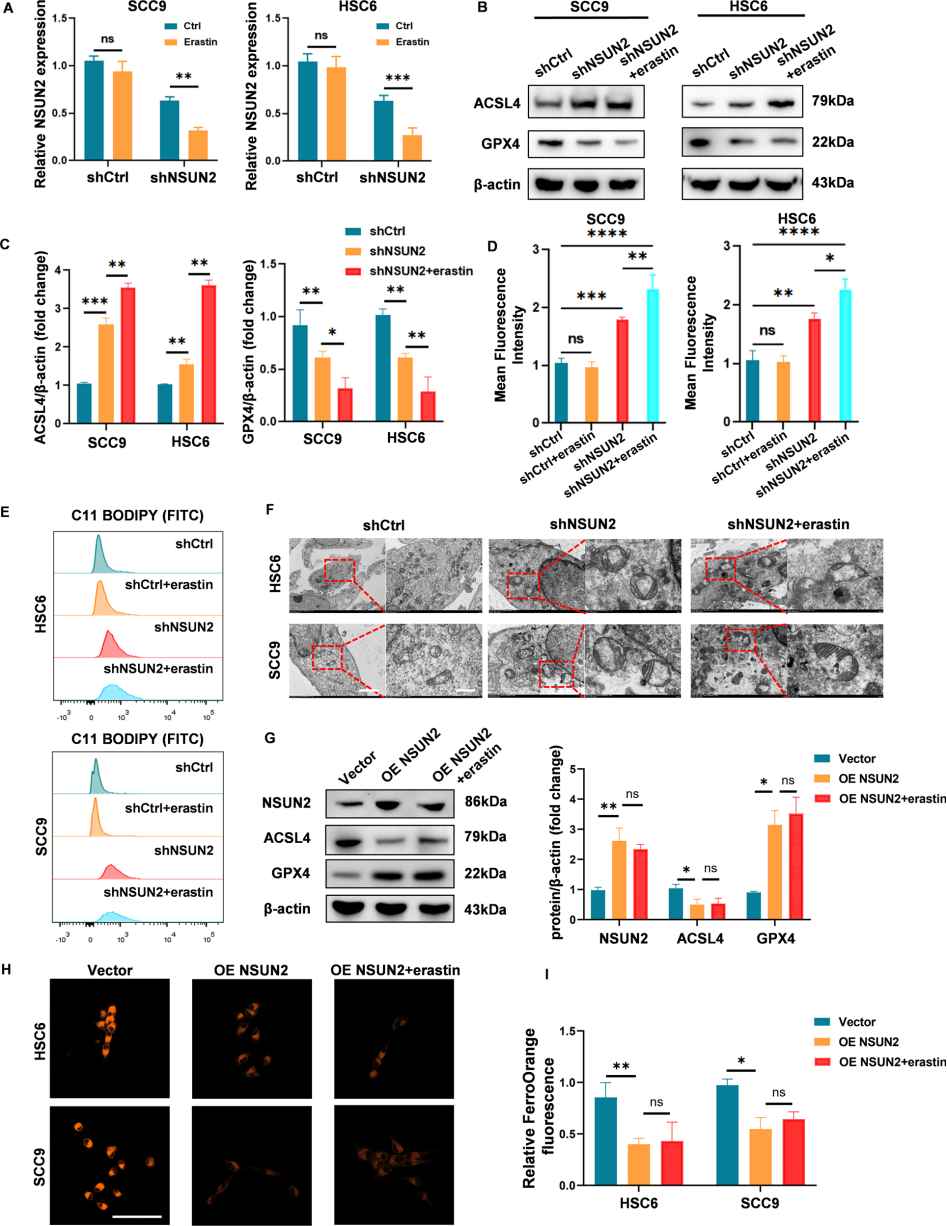
Knocking down and overexpression of NSUN2 regulates the sensitivity of OSCC cells to ferroptosis. SCC9 and HSC6 cells after transfection were treated with erastin. **A** The NSUN2 mRNA levels in SCC9 and HSC6 cells were detected by qRT-PCR. **B**, **C** The protein expression of ACSL4 and GPX4 in SCC9 and HSC6 cells transfected with NSUN2 shRNA were detected by western blotting. **D, E** Lipid peroxidation was measured via flow cytometry using BODIPY C11 581/591. **F** Representative TEM images of SCC9 and HSC6 cells. Scale bar: left = 2 μm, right = 500 nm. **G** The protein expression of ACSL4 and GPX4 in SCC9 and HSC6 cells transfected with OE-NSUN2 plasmid were detected by western blotting. **H**, **I** Representative IF images of the FerroOrange probe in SCC9 and HSC6 cells. Scale bar: 50 μm. *P < 0.05, **P < 0.01, ***P < 0.001, and ****P < 0.0001.

In two NSUN2-high expressed cell lines (SCC9 and HSC6), we knocked down NSUN2 to further clarify whether the suppression of NSUN2 may regulate sensitivity to ferroptosis in OSCC. Erastin treatment resulted in a 50% reduction in NSUN2 levels in cells with NSUN2 knockdown, but the NSUN2 levels in the control group (shCtrl) did not change (Fig. 3A). Western blot analysis revealed that NSUN2 knockdown reduced the expression of the ferroptosis marker GPX4 and transferrin and increased the expression of ACSL4 and SLC11A2. These effects were improved upon the addition of erastin (Fig. 3B, C, Fig. S2A, B). Additionally, transfection with shNSUN2 significantly increased lipid peroxidation (Fig. 3D, E) in erastin-treated SCC9 and HSC6 cells. Further investigation via TEM revealed that NSUN2-knockdown OSCC cells presented characteristic ferroptotic morphological changes, such as reduced mitochondria with increased membrane density. This phenomenon manifested with increased prominence together with the addition of erastin (Fig. 3F). Furthermore, we performed gain-of-function experiments by overexpressing NSUN2 in SCC9 and HSC6 cells using an OE-NSUN2 plasmid. Western blot analysis demonstrated that NSUN2 overexpression significantly elevated NSUN2 and GPX4 levels while decreasing ACSL4 expression, even in the presence of erastin (Fig. 3G, H). Consistent with this, immunofluorescence staining using the FerroOrange probe, which detects labile iron accumulation, revealed that NSUN2-overexpressing cells exhibited markedly reduced iron accumulation compared to control cells, even after erastin treatment (Fig. 3H, I). Taken together, our findings suggest that NSUN2 is essential for mediating ferroptosis resistance in OSCC and that its inhibition could increase the susceptibility of cancer cells to the induction of ferroptosis.

### NSUN2 mediates ferroptosis resistance in OSCC via m5C-dependent stabilization of SQSTM1/P62 mRNA

**Fig. 4:**
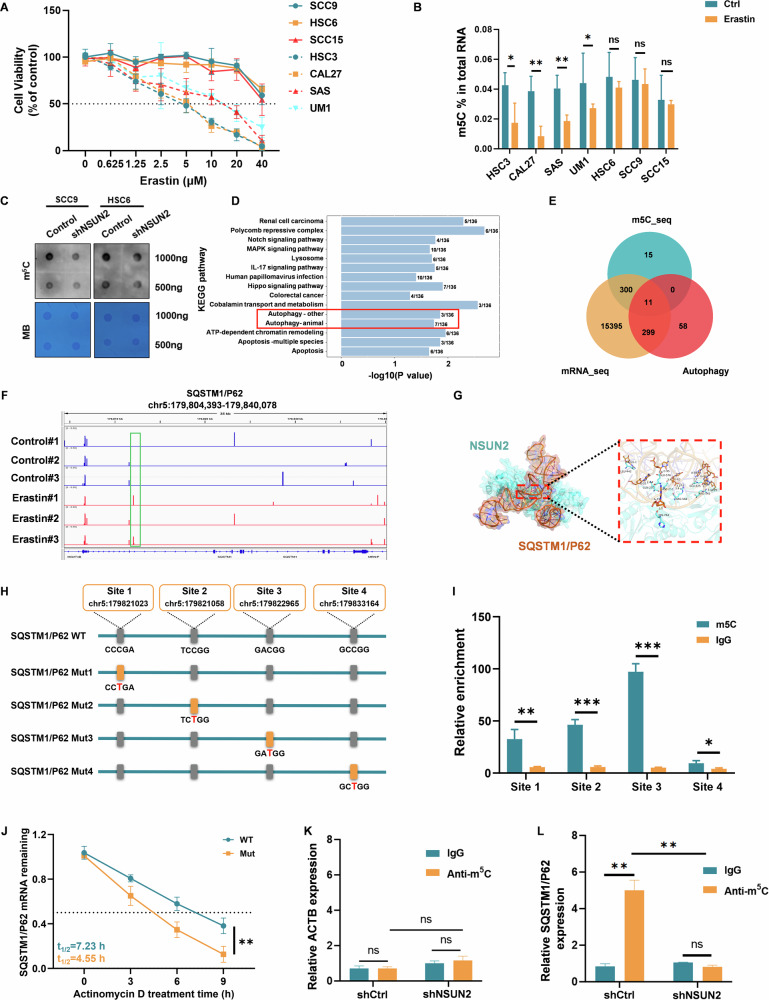
NSUN2 regulates autophagy in OSCC in an m5C-dependent manner by increasing m5C modification and stability of SQSTM1/P62 mRNA. **A** Cell viability was assessed via the CCK8 test after SCC9, HSC6, SCC15, HSC3, CAL27, SAS, and UM1 cells were exposed to the specified erastin doses for 24 h. **B** Levels of m5C in various cells following erastin addition. **C** The tendency for RNA m5C hypomethylation in cells with stable NSUN2 knockdown was detected via dot blot analysis. **D** Differentially expressed m5C genes related to ferroptosis in OSCC were extracted through RNA-seq. After KEGG analysis, the pathways enriched by the genes modified with DMC in the experimental group compared with the control group were shown. **E** Venn diagram illustrating the intersection of erastin downstream targets on the basis of genes from the autophagy pathway, m5C-seq, and RNA-seq. **F** Cells treated with or without erastin exhibit SQSTM1/P62 mRNA m5C sites. **G** Molecular docking analysis of NSUN2 and SQSTM1/P62 mRNA. **H** Plasmids with either wild-type or mutant (C → T mutation) m5C sites were constructed. The pattern diagram was shown. **I** Above constructed plasmid was transfected into stable NSUN2-overexpressing cells. The m5C modification of SQSTM1/P62 mRNA via MeRIP-qPCR in HSC6 cells were evaluated. **J** Residual mRNA levels of SQSTM1/P62 mRNA after termination of transcription via actinomycin D treatment in wild-type or mutant SQSTM1/P62 HSC6 cells. **K**, **L** SQSTM1/P62 mRNA relative IgG or anti-m5C antibody enrichment in HSC6 cells transfected with shNSUN2 or shCtrl. An m5C-specific antibody or an IgG control was used for the RIP tests. The negative control in this experiment was ACTB. MB, methylene blue; ACTB, β-actin. actinomycin D, 5 µg/ml. *P < 0.05, **P < 0.01, and ***P < 0.001.

Building on our prior demonstration that NSUN2 knockdown enhances ferroptosis sensitivity in OSCC cells, we next sought to dissect the molecular mechanisms linking NSUN2 to ferroptosis regulation. First, we evaluated the sensitivity of seven OSCC cell lines to erastin, a canonical ferroptosis inducer. Four lines (CAL27, SAS, UM1, HSC3) exhibited dose-dependent growth inhibition, whereas three lines (SCC9, HSC6, SCC15) remained resistant (Fig. 4A). Strikingly, resistant cells displayed significantly higher global m5C methylation levels compared to susceptible cells, and this methylation was unaltered by erastin treatment (Fig. 4B). In contrast, susceptible cells showed a notable reduction in m5C levels upon erastin exposure. Given NSUN2’s role as an m5C methyltransferase, we hypothesized that its activity might underlie this differential methylation. Indeed, knocking down NSUN2 in resistant cells (SCC9 and HSC6) recapitulated the m5C hypomethylation phenotype observed in susceptible cells (Fig. 4C), mirroring our prior finding that NSUN2 depletion enhances ferroptosis sensitivity.

Next, we supplemented the top 30 ferroptosis-related genes co-expressed with NSUN2 (Pearson correlation) from the ferroptosis gene database FerrDb (Fig. S3A). Common ferroptosis marker genes such as GPX4 and ACSL4 were not among them. Meanwhile, correlation analysis based on the TCGA database also verified that NSUN2 was not associated with GPX4, ACSL4, and Transferrin (Fig. S3B–D). This inspired us to further explore the intermediate link through which NSUN2 regulates ferroptosis in an m5C-dependent manner.

To explore possible NSUN2 targets in OSCC, we examined m5C-Bs-Seq and mRNA sequencing (mRNA-seq) data. We performed functional enrichment analysis on genes with differentially methylated CpG modifications in the experimental group compared to the control group, and the results were enriched in the autophagy pathway (Fig. 4D). To explore possible NSUN2 targets in the autophagy pathway in OSCC, we took the intersection of the screened differential genes with autophagy-related genes. Interestingly, SQSTM1/P62 and the related m5C alteration sites were shown to be important downstream targets of NSUN2 (Fig. 4E, F). Based on m5C-Bis-Seq data and NSUN2’s substrate preference (CG-rich motifs and CNGGG sequences), we identified four candidate m5C sites on SQSTM1/P62 mRNA: Site 1 (chr5:179821023), Sites 2 (chr5:179821058), Site 3 (chr5:179822965), and Site 4 (chr5:179833164). Molecular dynamics simulations demonstrated stable binding between NSUN2 and SQSTM1/P62 mRNA at the predicted sites (Fig. 4G). These interactions suggest NSUN2’s structural capacity to recognize and methylate SQSTM1/P62 mRNA at specific residues.

To confirm the functional relevance of these sites, we generated C → T mutations in the four regions (Sites 1-4) and tested their impact (Fig. 4H). The m5C modification of SQSTM1/P62 mRNA was evaluated via MeRIP-qPCR in HSC6 cells, which showed that m5C enrichment in the site 3 of SQSTM1/P62 mRNA was the most significant region (Fig. 4I). Furthermore, in NSUN2-overexpressing cells, wild-type SQSTM1/P62 mRNA exhibited a prolonged half-life, whereas mutant (C → T) SQSTM1/P62 mRNA showed no significant change (Fig. 4J). Anti-m5C antibody enriched SQSTM1/P62 mRNA by 5.0-fold in shCtrl cells (P < 0.01), whereas this enrichment was abolished in shNSUN2 cells (Fig. 4K, L). Furthermore, patients with high SQSTM1/P62 expression had much worse overall survival than those with low SQSTM1/P62 expression (Fig. S4A). In OSCC, SQSTM1/P62 mRNA expression was significantly upregulated in tumor tissues compared with their matched adjacent normal controls (Fig. S4B). Above experiments provided robust evidence that NSUN2 directly targets SQSTM1/P62 mRNA via m5C methylation, stabilizing its expression in OSCC.

### YBX1 mediates ferroptosis resistance in OSCC by enhancing NSUN2-dependent stabilization of SQSTM1/P62 mRNA

**Fig. 5:**
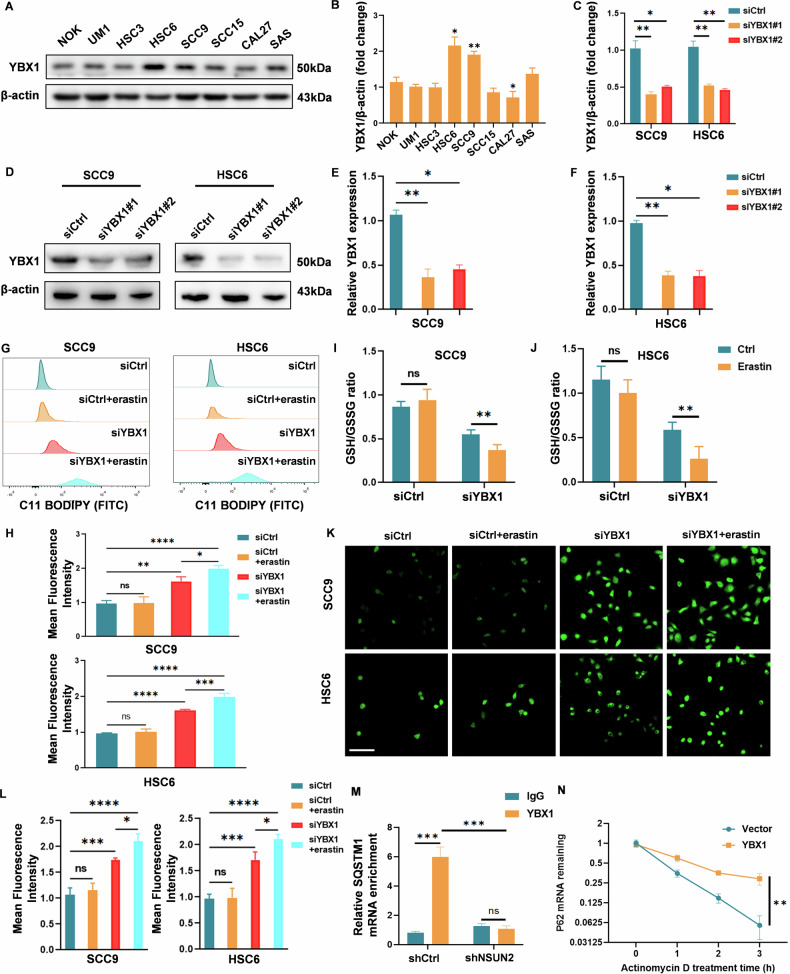
The m5C reader YBX1 is required for ferroptosis resistance. SCC9 and HSC6 cells were transfected with siRNA (siYBX1 or siCtrl) with or without erastin. **A**, **B** YBX1 protein expression were measured using western blotting in NOK, UM1, HSC3, HSC6, SCC9, SCC15, CAL27, and SAS cells, respectively. The protein **C**, **D** and mRNA **E**, **F** levels of YBX1 in SCC9 and HSC6 cells with YBX1 knockdown or the siCtrl control were determined via western blotting and qRT‒PCR, respectively. **G**, **H** Lipid peroxidation was measured by flow cytometry after C11BODIPY staining in SCC9 and HSC6 cells. **I**, **J** The relative GSH:GSSG ratios in SCC9 and HSC6 cells transfected with siRNA for 48 h were detected via GSH and GSSG, respectively. **K**, **L** ROS were detected by a fluorescent probe after transfection, with or without erastin treatment. Scale bar: 100 μm. **M** SQSTM1/P62 mRNA relative RIP enrichment ratio in HSC6 cells transfected with either shNSUN2 or shCtrl. YBX1-specific or IgG-specific antibodies were used for RIP-PCR. **N** SQSTM1/P62 residual mRNA levels in YBX1-overexpressing or vector control HSC6 cells following transcription termination with actinomycin D treatment. erastin: 10 μM; actinomycin D, 5 µg/ml. *P < 0.05, **P < 0.01, ***P < 0.001, and ****P < 0.0001.

The effect of the m5C reader YBX1 on ferroptosis resistance was then investigated to obtain more methodical knowledge of the role of aberrant m5C hypermethylation in ferroptosis resistance in OSCC. First, Kaplan–Meier survival analysis revealed that patients with high YBX1 expression exhibited significantly poorer overall survival compared to those with low YBX1 expression (Fig. S5A). Additionally, in OSCC, YBX1 mRNA levels were significantly upregulated in tumor tissues relative to their matched adjacent normal tissues (Fig. S5B). Similar to NSUN2, western blotting analysis further demonstrated that YBX1 expression was significantly higher in certain OSCC cell lines (HSC6 and SCC9) compared to NOK cells, whereas no statistically significant changes were observed in other OSCC cell lines (Fig. 5A, B). The effect of YBX1 knockdown on ferroptosis resistance was then investigated. The transfection efficiency was verified by western blot analysis and qRT‒PCR (Fig. 5C–F). Like NSUN2 knockdown, YBX1 silencing simultaneously increased lipid peroxidation (Fig. 5G, H) and markedly decreased the GSH/GSSG ratio in erastin-treated SCC9 and HSC6 cells (Fig. 5I, J), while also elevating ROS levels (Fig. 5K, L). Meanwhile, RIP‒PCR revealed that the knockdown of NSUN2 resulted in a marked decrease in the enrichment of SQSTM1/P62 mRNA by YBX1 (Fig. 5M). Similarly, to assess the impact of YBX1 on the stability of SQSTM1/P62 mRNA, we treated YBX1-overexpressing cells with the RNA synthesis inhibitor actinomycin D. The results demonstrated that YBX1 overexpression enhances the stability of SQSTM1/P62 mRNA (Fig. 5N). These findings suggest that the silencing of YBX1 not only induces ferroptosis but also enhances the response to ferroptosis inducers such as erastin in previously resistant OSCC cells, highlighting the critical role of YBX1 in maintaining ferroptosis resistance. Collectively, these results suggest that NSUN2 regulates the expression and stability of SQSTM1/P62 through YBX1.

### NSUN2 Mediated SQSTM1/P62 Upregulation, Suppressing Autophagy and Ferroptosis Resistance in OSCC

**Fig. 6:**
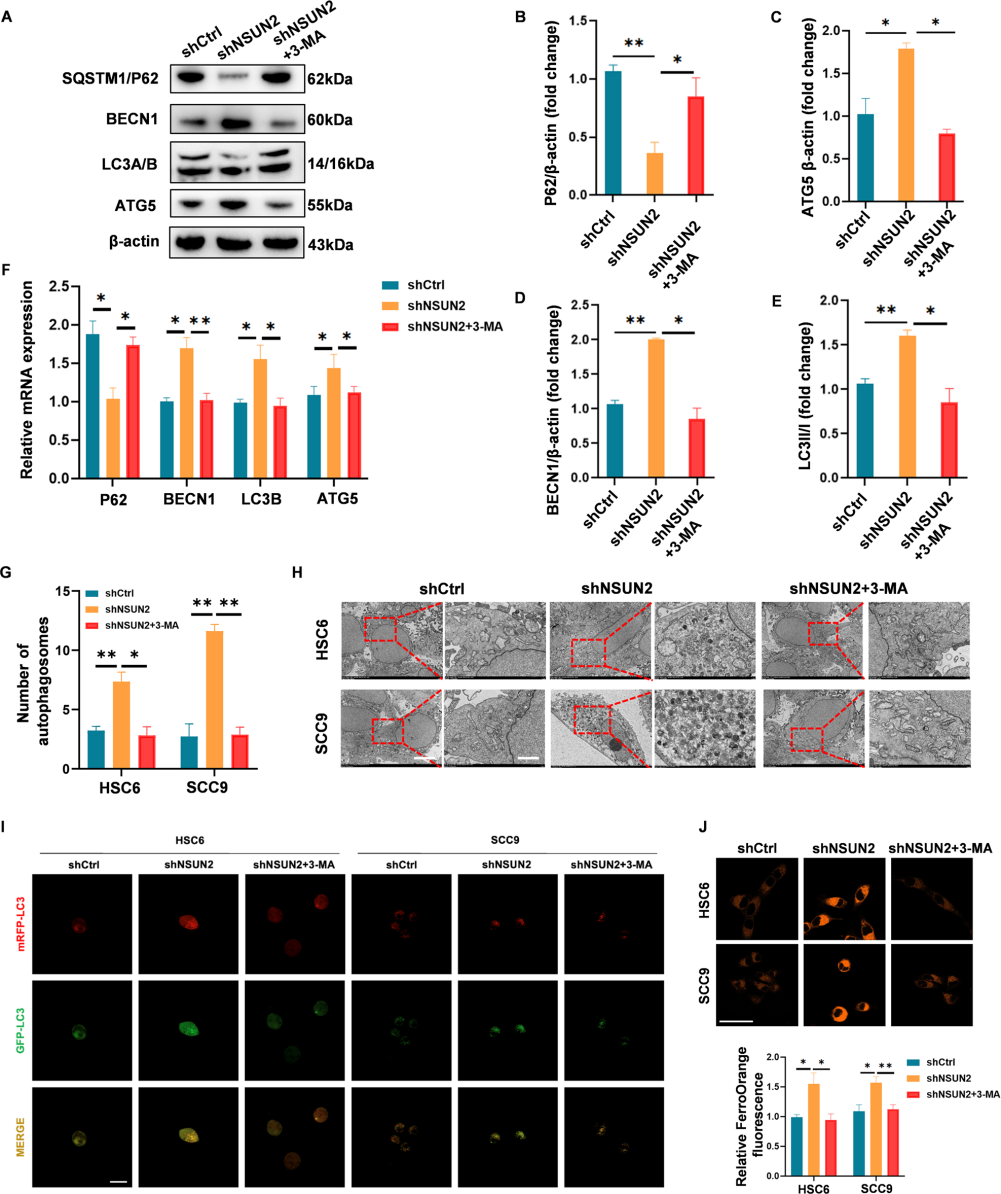
Autophagy suppression and subsequent ferroptosis resistance in OSCC are caused by NSUN2-mediated SQSTM1/P62 upregulation. SCC9 and HSC6 cells were transfected with shNSUN2 with or without 3-MA treatment. western blotting **A‒E** and qRT‒PCR **F** of the autophagy markers SQSTM1, BECN1, LC3A/B and ATG5 in HSC6 cells. **G**, **H** Representative TEM images and quantitative analysis of SCC9 and HSC6 cells. **I** Representative images of shNSUN2, shNSUN2 + 3-MA and control cells after transfecting with mRFP-GFP-LC3 lentivirus. Scale bars, 20 μm. **J** Representative IF images of the FerroOrange probe in SCC9 and HSC6 cells. Scale bar: 50 μm. 3-MA: 1 mM. *P < 0.05, and **P < 0.01.

In the preceding results, we identified SQSTM1/P62 as a critical downstream target of NSUN2-mediated m5C methylation, whereby NSUN2 stabilizes SQSTM1/P62 mRNA to sustain its expression. We next sought to explore the functional implications of this regulatory axis in autophagy. Western blot analysis and qRT-PCR confirmed the changes in the expression of autophagy markers. The decrease in SQSTM1/P62 and the increase of ATG5, BECN1, and the ratio of LC3B-I to LC3B-II suggested that NSUN2 knockdown promoted autophagy, which 3-MA could successfully block (Fig. 6A–F). In line with the overexpression of autophagy-related proteins, TEM images revealed a notable increase in the quantity and size of autophagosomes. Treatment with 3-MA led to a remarkable reduction in the number and morphology of autophagosomes (Fig. 6G, H). Knockdown of NSUN2 resulted in a significant decrease in mRFP-GFP-LC3 puncta accumulation, whereas treatment with 3-MA yielded the opposite effect, indicating that NSUN2 downregulation may promote autophagosome formation (Fig. 6I).

To investigate whether 3-MA affects ferroptosis, intracellular Fe^2+^ was detected with a FerroOrange fluorescent probe (Fig. 6J). Knockdown of NSUN2 increased intracellular iron accumulation in SCC9 and HSC6 cells. However, when 3-MA was added, the fluorescence intensity significantly decreased, suggesting that the ferroptosis induced by NSUN2 knockdown was rescued by the autophagy inhibitor 3-MA. These findings suggest that NSUN2 may regulate susceptibility to ferroptosis in OSCC through its effects on autophagy, highlighting the complex interplay between these two cellular processes in the context of cancer biology.

### NSUN2 knockdown suppresses OSCC tumorigenesis via autophagy-dependent ferroptosis in vivo

**Fig. 7:**
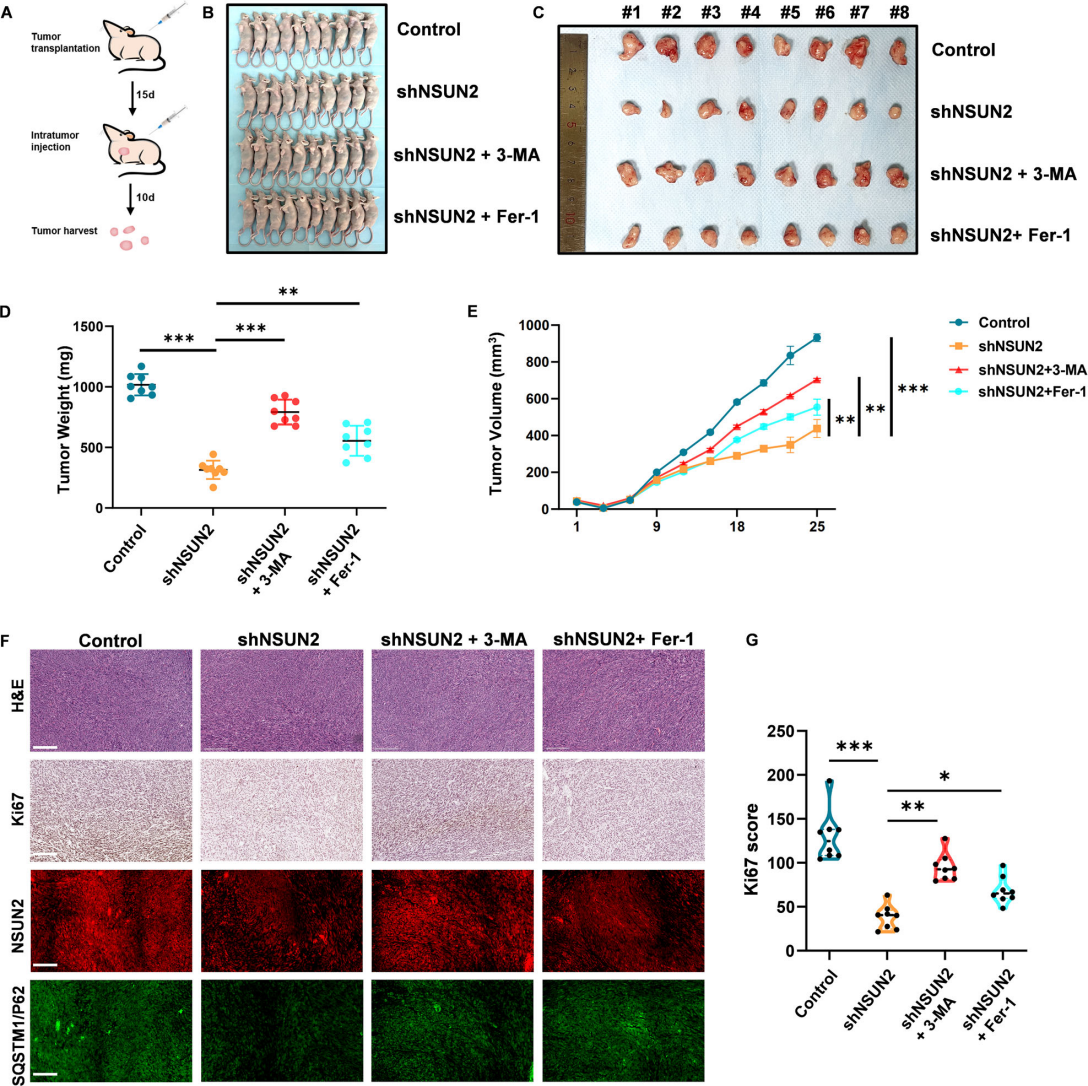
The addition of 3-MA or Fer-1 partly rescued the tumorigenesis inhibited by NSUN2 knockdown in vivo. **A** Specific strategy and timeline of the animal experiments. **B**, **C** Tumors extracted from nude mice subjected to different treatments are shown (n = 8 per group). **D** Tumors were weighed after the mice were sacrificed. **E** Tumor volume was measured every 3 days. **F** Representative images of H&E staining, Ki67 IHC staining, and IF staining of NSUN2 and SQSTM1/P62 in tumors from NSUN2-knockdown HSC6 cells, with or without inhibitors. Scale bars: 100 µm. **G** Ki67 score of **F**. *P < 0.05, **P < 0.01, and ***P < 0.001.

**Fig. 8:**
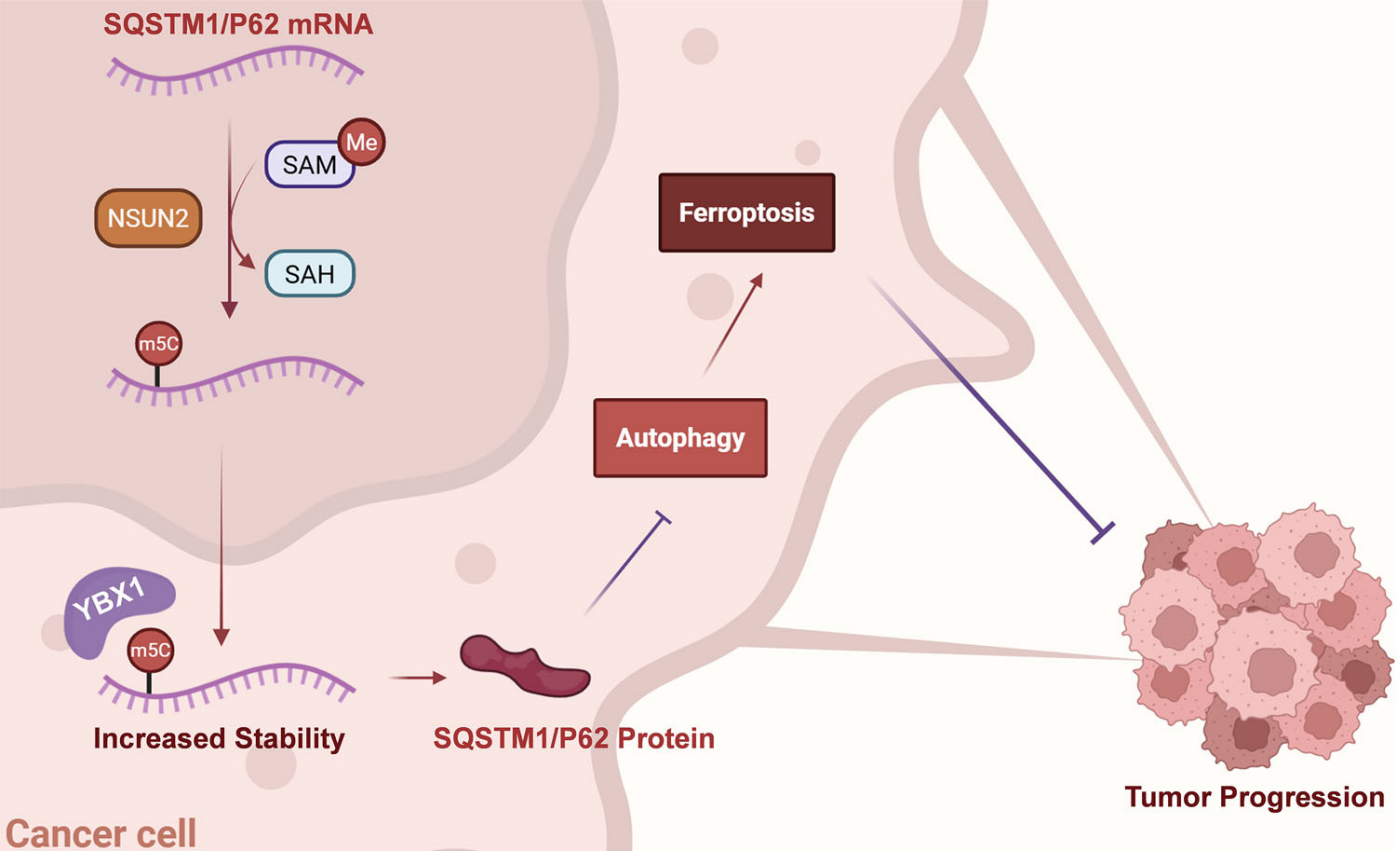
The speculative mechanism diagram.

We next explored this NSUN2-autophagy-ferroptosis regulatory axis in vivo using a nude mouse xenograft model (Fig. 7A). NSUN2 knockdown significantly reduced the tumorigenicity of HSC6 cells in vivo, which is in line with our findings in vitro. Additionally, 3-MA treatment partially restored tumor growth in shNSUN2 cells, indicating autophagy is critical for NSUN2 loss-induced tumor suppression. Fer-1 treatment also partially rescued tumor growth, demonstrating ferroptosis contributes to the phenotype (Fig. 7B–E). These data mechanistically link NSUN2 loss to autophagy-dependent ferroptosis in vivo. Immunohistochemistry revealed that the expression of Ki67 decreased after NSUN2 knockdown but was restored upon treatment with 3-MA or Fer-1 (Fig. 7F, G). In vivo, NSUN2 and SQSTM1/P62 expression was positively correlated, according to IF analysis of tumor sections. This relationship was reversed after 3-MA or Fer-1 treatment (Fig. 7F). Collectively, these in vivo results validate our in vitro findings, underscoring the critical role of the NSUN2-SQSTM1/P62 axis in regulating autophagy and ferroptosis to promote OSCC tumor growth (Fig. 8).

## Discussion

For decades, the mortality rate of OSCC has remained constant, making it one of the world’s leading causes of cancer-related deaths [1]. The development of efficient treatment strategies is urgently needed. The clinical progression and poor prognosis of OSCC were found to be substantially correlated with NSUN2 overexpression in this study. In OSCC cells resistant to ferroptosis, knockdown of NSUN2 expression remarkably enhanced their sensitivity to ferroptosis and inhibited the proliferation of OSCC cells. Mechanistically, increased NSUN2 expression elevated the m5C modification of SQSTM1/P62 mRNA, thereby suppressing ferroptosis in OSCC by regulating autophagy. The m5C reader protein YBX1 improved the stability of SQSTM1/P62. Therefore, NSUN2 is a key gene that mediates the regulation of ferroptosis sensitivity in OSCC through m5C methylation and is a potential therapeutic target for OSCC.

Post-transcriptional RNA modifications are widely involved in tumorigenesis and tumor progression [33, 34]. Based on bioinformatics, a high m5C score in OSCC patients has been linked to a significantly lower overall and progression-free survival rate as well as a higher tumor recurrence rate. This suggests that m5C is an important epigenetic modification of RNA and can be used as a new and accurate prognostic model [35, 36]. NSUN2 is one of the m5C methyltransferases that has attracted the most attention because of its carcinogenic potential in a variety of cancer types [30, 37–41]. Our results also identified high expression of NSUN2 in multiple OSCC clinical samples and OSCC cells, which was correlated with poor prognosis. To our knowledge, there have been no reports on the mechanism of m5C modification in OSCC. In addition to the correlation between NSUN2 expression and T cell activation status influencing the survival rate of HNSC patients [42], NSUN2 can increase the malignant phenotype of HNSC cells through its methyltransferase function [43]. This study is the first to focus on how NSUN2 changes the m5C methylation and leads to the development of cancer in OSCC. Our findings showed that OSCC carcinogenesis and metastasis could be considerably reduced by NSUN2 downregulation.

Apoptosis, ferroptosis, necroptosis, autophagic cell death and necroptosis are among the several regulated cell death mechanisms that may be induced to treat a variety of cancers. Every regulated cell death activity has its own subroutines and is subject to modulation by distinct signal transduction pathways [44, 45]. Thus, it is very promising to find therapeutic targets and gain insights into novel cancer treatment techniques by revealing the precise molecular processes that underlie their regulatory networks. Ferroptosis is a new type of controlled cell death brought on by the buildup of lipid peroxides that are dependent on iron [29, 46]. It is essential for controlling the development and spread of certain cancer cell types, including as ovarian, gastric, hepatic, and colorectal cancers [47]. Targeting ferroptosis in tumor cells appears to be a very promising therapeutic approach, according to growing data. Reversing cancer resistance to popular treatments including chemotherapy, targeted therapy, and immunotherapy requires modifying ferroptosis [48–50]. Ferroptosis is strictly controlled at several levels, including as the transcriptional, post-transcriptional, epigenetic, and post-translational layers [29]. Although many cancer cells are naturally resistant to ferroptosis inducers, these substances might be novel targets for cancer treatment [51]. In this research, we found that HSC6, SCC9, and SCC15 cells exhibited low sensitivity to erastin among 7 OSCC cell lines. Ferroptosis resistance promotes the tumorigenic and metastatic abilities of tumors. We observed that HSC6 and SCC9 cells with high NSUN2 expression were more resistant to the ferroptosis inducer erastin than other OSCC cell lines (including HSC3, CAL27, SAS, and UM1 cells). Additionally, we found that the difference in NSUN2 protein levels between these two cell lines appeared to be consistent with the difference in erastin sensitivity. Further experiments revealed that in ferroptosis-insensitive cells, NSUN2 knockdown reduced the hypermethylation level of HSC6 and SCC9 cells and restored sensitivity to the ferroptosis inducer erastin, suggesting that NSUN2 may be a major factor in OSCC ferroptosis resistance. This is consistent with previous reports that the high methylation state of mRNA and mitochondrial RNA driven by the M5C methyltransferase NSUN2 induces ferroptosis resistance and promotes tumorigenesis [29, 31].

The multi-domain protein known as SQSTM1/P62 interacts with the autophagy process and serves as a crucial adapter protein for target proteins. It uses its LC3-interacting domain to engage with phagocytes, its ubiquitin-associated domain to directly bind to ubiquitinated protein aggregates, and its PB1 domain to sequester them into inclusion bodies [52]. This protein acts as an anchor for interactions with a number of important signal transduction proteins. Recent data points to SQSTM1/P62’s effect on causing oncogenic transformation in a variety of cells [52–54]. Autophagy insufficiency has been shown to cause chronic SQSTM1/P62 expression, which is enough to change NF-κB regulation and gene expression and encourage the growth of tumors [55]. In fact, treatment resistance, tumor development, and cancer promotion are linked to SQSTM1/P62 overexpression and/or decreased degradation [52]. In cancer, the expression level of SQSTM1/P62 is often correlated with tumor progression and prognosis. SQSTM1/P62 is upregulated in colorectal cancer tissues [56]. Overall survival and disease-free survival in ovarian cancer are significantly correlated negatively with SQSTM1/P62 expression [57]. SQSTM1/P62 functions as a receptor protein for the autophagic degradation of ubiquitinated proteins. It can attach to ubiquitinated proteins for transport to autophagic degradation and, by removing ubiquitinated proteins, contribute to cisplatin resistance in human ovarian cancer cells [58].

A growing number of studies have elucidated the role of epigenetic regulation of autophagy in modulating cancer. Modifications such as acetylation, phosphorylation, and methylation can affect autophagy. For instance, autophagy-dependent senescence in HNSC was used to overcome the radioresistance of head and neck cancer cells by the epigenetic control of SQSTM1/P62 [59]. Furthermore, in diabetic skin, the m6A reader YTHDC1 targeted SQSTM1/P62 to control autophagy [29]. Our study was the first to clarify that m5C methylation modification on the autophagy receptor protein SQSTM1/P62 can promote OSCC tumors. Recent studies have indicated that cancer cells can utilize the autophagy mechanism to trigger ferroptosis [60–62], potentially suggesting a new approach for inhibiting tumor development through the autophagy pathway. Currently, there are two mechanisms by which SQSTM1/P62 regulates ferroptosis: the SQSTM1/P62/Keap1/Nrf2 pathway [63–65] and the autophagy receptor SQSTM1/P62, acting as a platform to support the interaction between NCOA4 and LC3, which promotes ferritin degradation and increases ferroptosis sensitivity [66]. According to our research, NSUN2 led to the m5C methylation alteration on SQSTM1/P62 mRNA. Additionally, the presence of the reader YBX1 enhanced SQSTM1/P62 stability, which in turn prevented ferroptosis and autophagy. This study reveals another way in which SQSTM1/P62 regulates ferroptosis, and further research is needed to determine the potential mechanism by which SQSTM1/P62 regulates autophagy-dependent ferroptosis in OSCC.

Future studies could focus on developing more physiologically relevant models to better understand the role of NSUN2 in OSCC. This would enable a more accurate representation of the complex tumor microenvironment of human OSCC, which was a limitation of the current study. Additionally, it is essential to further explore the molecular mechanisms underlying the interaction between NSUN2 and other epigenetic regulators. Investigating the potential crosstalk between m5C methylation and other RNA modifications in modulating ferroptosis and tumor progression could also provide valuable insights.

## Supplementary information

Supplemental material

wb original data

## Data Availability

The datasets supporting the conclusions of this article are available in the GSA repository (https://ngdc.cncb.ac.cn/gsa-human/).(Accession Number: HRA011481).
