# Supplementary material for: NSUN2 mediated-aberrant 5-methylcytosine methylation regulates autophagy-related ferroptosis in oral squamous cell carcinoma progression
Source: Cell Death Dis. 2025 Dec 23;16(1):903. doi: 10.1038/s41419-025-08174-y (PMC12728175; doi:10.1038/s41419-025-08174-y)
Supplement: Supplementary file 1 — Supplemental material [file 41419_2025_8174_MOESM1_ESM.docx]

**Table S1.** clinicopathological characteristics stratified by NSUN2 expression in OSCC patients.

| **Variables** | **N (%)** | **High NSUN2** | **Low NSUN2** | ***P* value** |
| --- | --- | --- | --- | --- |
| **Age** (Year) |  |  |  |  |
| ≤ 60 | 141 (47.3) | 111 | 30 | 0.55 |
| > 60 | 157 (52.7) | 119 | 38 |  |
| **Gender** |  |  |  |  |
| Male | 202 (67.8) | 158 | 44 | 0.54 |
| Female | 96 (32.2) | 72 | 24 |  |
| **Grade** |  |  |  |  |
| G1 | 47 (16.0) | 34 | 13 | 0.51 |
| G2 | 186 (63.3) | 147 | 39 |  |
| G3 | 61 (20.7) | 45 | 16 |  |
| **Clinical T stage** |  |  |  |  |
| T1 / T2 | 112 (38.1) | 82 | 30 | 0.20 |
| T3 / T4 | 182 (61.9) | 145 | 37 |  |
| **Clinical N stage** |  |  |  |  |
| N0 | 159 (55.0) | 120 | 39 | 0.28 |
| N1 / N2 / N3 | 130 (45.0) | 105 | 25 |  |
| **Stage** |  |  |  |  |
| Stage I / Stage II | 75 (25.2) | 52 | 23 | 0.06 |
| Stage III / Stage IV | 223 (74.8) | 178 | 45 |  |

**Table S2.** **Primer sequences used in this study.**

| Gene | | Primer sequences（5'-3'） |
| --- | --- | --- |
| NSUN2-F | | GAACTTGCCTGGCACACAAAT |
| NSUN2-R | | TGCTAACAGCTTCTTGACGACTA |
| GPX4-F | | GAGGCAAGACCGAAGTAAACTAC |
| GPX4-R | | CCGAACTGGTTACACGGGAA |
| ACSL4-F | | ACTGGCCGACCTAAGGGAG |
| ACSL4-R | | GCCAAAGGCAAGTAGCCAATA |
| YBX1-F | | GGGGACAAGAAGGTCATCGC |
| YBX1-R | | CGAAGGTACTTCCTGGGGTTA |
| SQSTM1/P62-F | | GACTACGACTTGTGTAGCGTC |
| SQSTM1/P62-R | | AGTGTCCGTGTTTCACCTTCC |
| ATG5-F | | AAAGATGTGCTTCGAGATGTGT |
| ATG5-R | | CACTTTGTCAGTTACCAACGTCA |
| Beclin 1-F | | GAGCTAAAGGAGCTGGCACT |
| Beclin 1-R | | TCTGCCACTATCTTGCGGTT |
| LC3B-F | | GATGTCCGACTTATTCGAGAGC |
| LC3B-R | | TTGAGCTGTAAGCGCCTTCTA |
| β-actin-F | CTACCTCATGAAGATCCTCACCGA | |
| β-actin-R | | TTCTCCTTAATGTCACGCACGATT |

**Table S3. Sequences of shRNAs and siRNAs used in this study.**

| Name | Sequence (5’-3’) |
| --- | --- |
| shNSUN2#1 | ACCGGGAGCGATGCCTTAGGATATTATTCAAGAGATAATATCCTAAGGCATCGCTCTTTTTTGAATTC |
| shNSUN2#2 | ACCGGcagtggaaggtaatgacgaaactcgagtttcgtcattaccttccactgTTTTTTGAATTC |
| shCtrl | ACCGGGCGTGATCTTCACCGACAAGATTCAAGAGATCTTGTCGGTGAAGATCACGCTTTTTTGAATTC |
| OE-NSUN2 | GCTAGCGCCACCATGGGGCGGCGGTCGCGGGGTCGGCGGCTCCAGCAACAGCAGCGGCCGGAGGACGCGGAGGATGGCGCCGAGGGTGGTGGAAAGCGCGGCGAGGCGGGCTGGGAAGGAGGCTACCCCGAGATCGTCAAGGAGAACAAGCTGTTCGAGCACTACTACCAGGAGCTCAAGATCGTGCCCGAGGGCGAGTGGGGCCAGTTCATGGACGCTCTCAGGGAGCCGCTCCCGGCCACTTTAAGAATTACTGGTTACAAAAGCCACGCAAAAGAGATTCTCCATTGCTTAAAGAACAAATATTTTAAGGAATTGGAGGACCTGGAGGTGGACGGTCAGAAAGTTGAAGTTCCACAGCCACTGAGTTGGTATCCTGAAGAACTTGCCTGGCACACAAATTTAAGTCGAAAAATCTTGAGAAAATCGCCACACTTGGAAAAGTTTCATCAGTTTCTAGTTAGTGAAACAGAATCTGGAAATATTAGTCGTCAAGAAGCTGTTAGCATGATCCCACCACTGCTCCTCAACGTGCGGCCTCATCATAAGATCTTAGATATGTGTGCAGCACCTGGCTCAAAGACCACACAGTTAATTGAAATGCTACATGCCGACATGAATGTCCCCTTTCCAGAGGGATTTGTTATTGCGAATGATGTGGACAACAAGCGCTGCTACCTGCTCGTCCATCAAGCCAAGAGGCTGAGCAGCCCCTGCATCATGGTGGTCAACCATGATGCCTCCAGCATACCCAGGCTCCAGATAGATGTGGACGGCAGGAAAGAGATCCTCTTCTATGATCGAATTTTATGTGATGTCCCTTGCAGTGGAGACGGCACTATGAGAAAAAACATTGATGTTTGGAAAAAGTGGACCACCTTAAATAGCTTGCAGCTACATGGCTTACAGCTGCGGATTGCAACACGCGGGGCTGAACAGCTGGCTGAAGGTGGAAGGATGGTGTATTCCACGTGTTCACTAAACCCTATTGAGGATGAAGCAGTCATAGCATCTTTACTGGAAAAAAGTGAAGGTGCTTTGGAGCTTGCTGATGTGTCTAATGAACTGCCAGGGCTGAAGTGGATGCCTGGAATCACACAGTGGAAGGTAATGACGAAAGATGGGCAGTGGTTTACAGACTGGGACGCTGTTCCTCACAGCAGACACACCCAGATCCGACCTACCATGTTCCCTCCGAAGGACCCAGAAAAGCTGCAGGCCATGCACCTGGAGCGATGCCTTAGGATATTACCCCATCATCAGAATACTGGAGGGTTTTTTGTGGCAGTATTGGTGAAAAAATCTTCAATGCCGTGGAATAAACGTCAGCCAAAGCTTCAGGGTAAATCTGCAGAGACCAGAGAAAGCACACAGCTGAGCCCTGCAGATCTCACAGAAGGGAAACCCACAGATCCCTCTAAGCTGGAAAGTCCGTCATTCACAGGAACTGGTGACACAGAAATAGCTCATGCAACTGAGGATTTAGAGAATAATGGCAGTAAGAAAGATGGCGTGTGTGGTCCTCCTCCATCAAAGAAAATGAAGTTATTTGGATTTAAAGAAGATCCATTTGTATTTATTCCTGAAGATGACCCATTATTTCCACCTATTGAGAAATTTTATGCTTTGGATCCTTCATTCCCAAGGATGAATTTGTTAACTCGGACTACAGAAGGGAAGAAAAGGCAGCTCTACATGGTTTCTAAGGAGTTGCGGAATGTGCTGCTGAATAACAGTGAGAAGATGAAGGTTATTAACACGGGGATCAAAGTCTGGTGTAGAAATAACAGCGGTGAAGAGTTTGACTGTGCTTTCCGGCTGGCACAGGAGGGAATATATACATTGTATCCATTTATTAACTCAAGAATTATTACTGTATCAATGGAAGATGTTAAGATACTGTTGACCCAGGAAAATCCCTTTTTTAGAAAACTCAGCAGTGAGACCTACAGTCAAGCAAAGGACCTGGCAAAGGGAAGCATCGTGCTGAAGTATGAACCAGATTCTGCGAATCCAGACGCTCTGCAGTGTCCCATCGTCTTATGCGGATGGCGGGGAAAGGCCTCCATTCGAACTTTTGTGCCCAAGAATGAACGGCTTCATTATCTCAGGATGATGGGGCTGGAGGTATTGGGAGAAAAGAAGAAGGAAGGGGTTATCCTCACAAATGAGAGTGCAGCCAGCACCGGACAGCCAGACAATGACGTGACTGAGGGACAGAGAGCAGGAGAGCCCAACAGCCCAGATGCAGAAGAGGCCAACAGTCCAGACGTGACAGCAGGCTGTGACCCGGCGGGGGTCCATCCACCCCGGTGAGCGGCCGC |
| siCtrl | siN0000001-1-5 |
| siYBX1#1 | UUUGCUGGUAAUUGCGUGGAGGACC |
| siYBX1#2 | UAUUUCUUCUUGUUGGAUGACUAAA |
| OE-YBX1 | TCTAGAgccaccatgagcagcgaggccgagacccagcagccgcccgccgccccccccgccgcccccgccctcagcgccgccgacaccaagcccggcactacgggcagcggcgcagggagcggtggcccgggcggcctcacatcggcggcgcctgccggcggggacaagaaggtcatcgcaacgaaggttttgggaacagtaaaatggttcaatgtaaggaacggatatggtttcatcaacaggaatgacaccaaggaagatgtatttgtacaccagactgccataaagaagaataaccccaggaagtaccttcgcagtgtaggagatggagagactgtggagtttgatgttgttgaaggagaaaagggtgcggaggcagcaaatgttacaggtcctggtggtgttccagttcaaggcagtaaatatgcagcagaccgtaaccattatagacgctatccacgtcgtaggggtcctccacgcaattaccagcaaaattaccagaatagtgagagtggggaaaagaacgagggatcggagagtgctcccgaaggccaggcccaacaacgccggccctaccgcaggcgaaggttcccaccttactacatgcggagaccctatgggcgtcgaccacagtattccaaccctcctgtgcagggagaagtgatggagggtgctgacaaccagggtgcaggagaacaaggtagaccagtgaggcagaatatgtatcggggatatagaccacgattccgcaggggccctcctcgccaaagacagcctagagaggacggcaatgaagaagataaagaaaatcaaggagatgagacccaaggtcagcagccacctcaacgtcggtaccgccgcaacttcaattaccgacgcagacgcccagaaaaccctaaaccacaagatggcaaagagacaaaagcagccgatccaccagctgagaattcgtccgctcccgaggctgagcagggcggggctgaggattacaaggatgacgacgataagtaaGCGGCCGC |

**
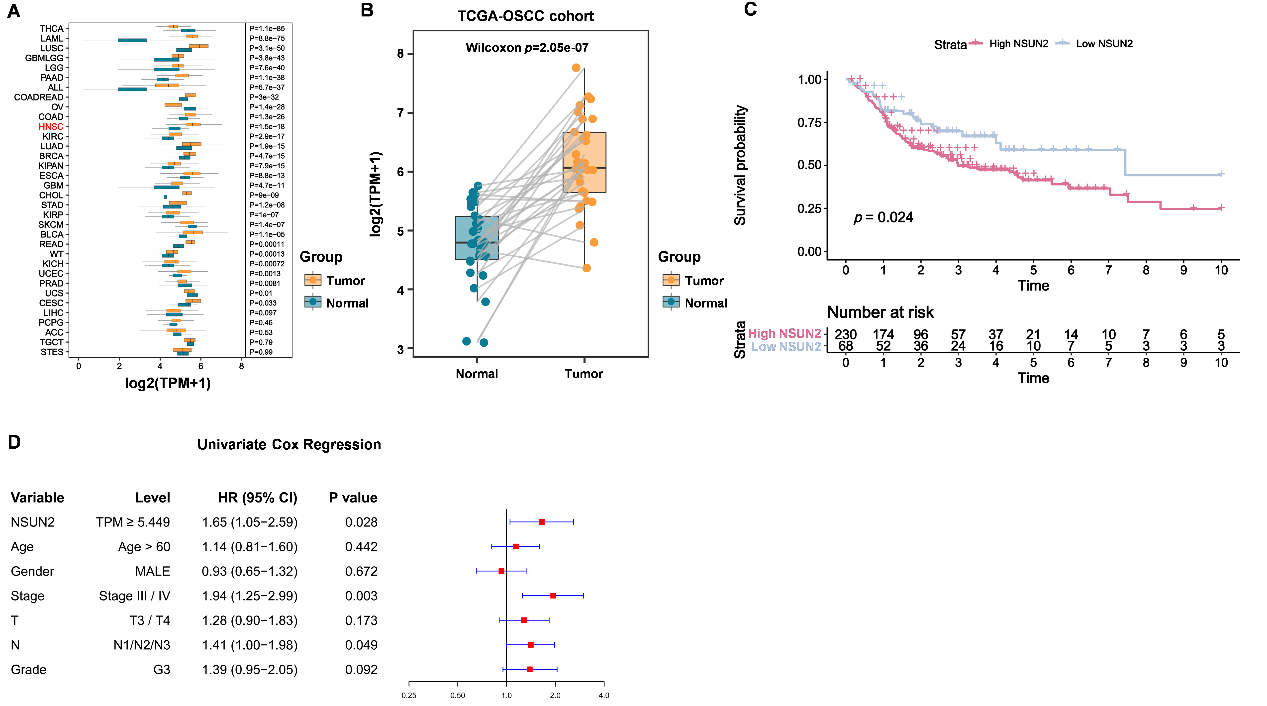
Figure S1. NSUN2 is upregulated in OSCC and predicts poor prognosis in TCGA datasets**

**(A)** Gene expression profiles of NSUN2 across pan-cancer datasets from the TCGA database. **(B)** Elevated NSUN2 expression in OSCC tissues versus adjacent normal tissues in the TCGA-OSCC cohort (n = 30). **(C)** Kaplan-Meier survival analysis of OSCC patients with high and low NSUN2 expression from the TCGA dataset (n = 298; generated via Kaplan-Meier Plotter). **(D)** Univariate Cox regression analyses of overall survival and clinicopathological features in OSCC patients.


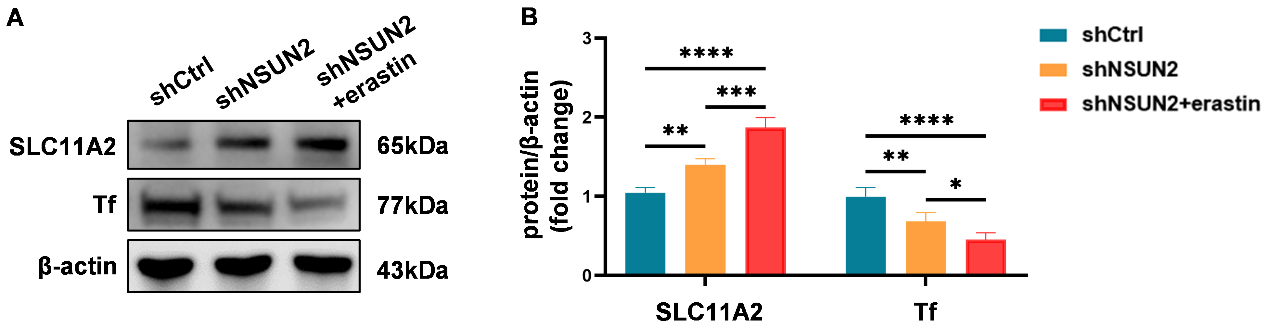
**Figure S2.** **NSUN2 knockdown enhances erastin-induced regulation of ferroptosis markers in OSCC cells**

**(A)** The protein expression of SLC11A2 and Transferrin in SCC9 and HSC6 cells transfected with NSUN2 shRNA were detected by western blotting, with or without treatment of erastin. **(B)** Quantitative analysis of (A). **P* < 0.05, ***P* < 0.01, ****P* < 0.001, and *****P* < 0.0001.


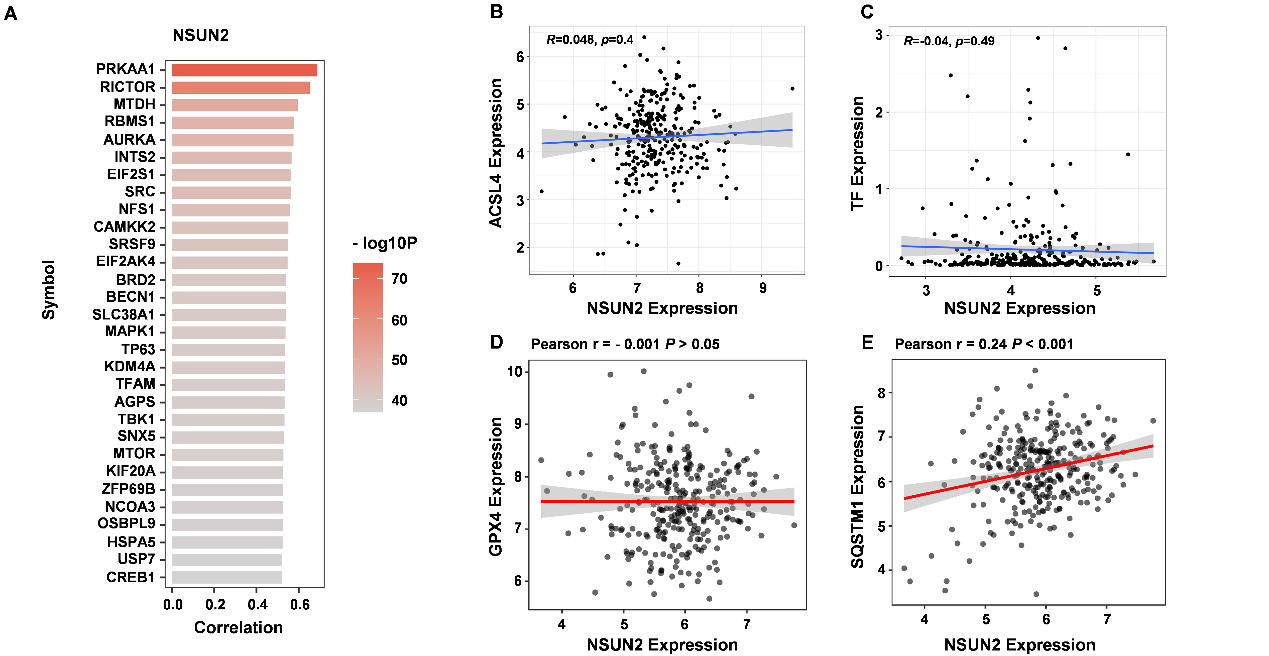
**Figure S3.** **Co-expression analysis of NSUN2 with ferroptosis-related genes and correlation validation in TCGA datasets**

(A) The top 30 ferroptosis-related genes from the ferroptosis gene database FerrDb co-expressed with NSUN2. Correlation between relative expression of NSUN2 and ACSL4 **(B)**, Tf **(C)**, GPX4 **(D)**, SQSTM1 **(E)** using Pearson correlation test based on TCGA dataset. Tf, Transferrin.


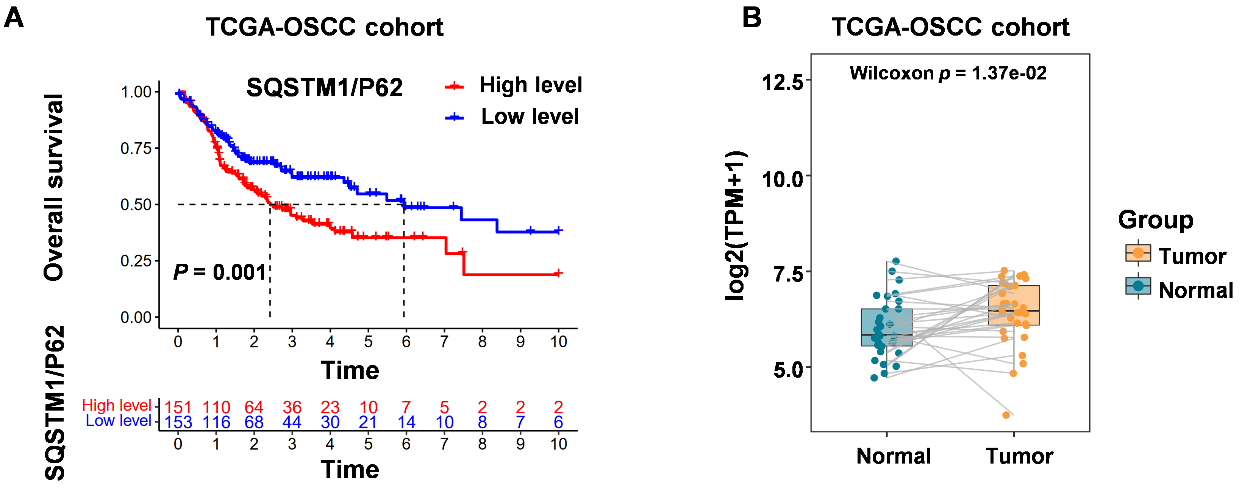
**Figure S4.** **Upregulated SQSTM1/P62 expression in OSCC tissues correlates with poor patient prognosis in TCGA datasets**

(A) Elevated SQSTM1/P62 expression in OSCC tissues versus adjacent normal tissues in the TCGA-OSCC cohort (n = 30). **(B)** Kaplan-Meier survival analysis of OSCC patients with high and low SQSTM1/P62 expression from the TCGA dataset (n = 298; generated via Kaplan-Meier Plotter).


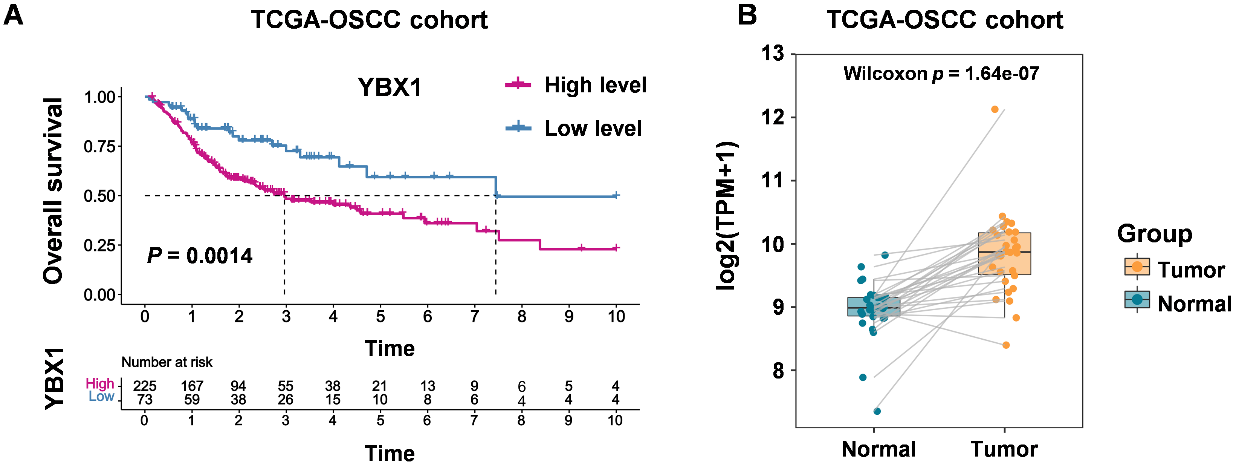
**Figure S5. Upregulated YBX1 expression in OSCC tissues correlates with poor patient prognosis in TCGA datasets**

**(A)** Elevated YBX1 expression in OSCC tissues versus adjacent normal tissues in the TCGA-OSCC cohort (n = 30). **(B)** Kaplan-Meier survival analysis of OSCC patients with high and low YBX1 expression from the TCGA dataset (n = 298; generated via Kaplan-Meier Plotter).
