# Supplementary figures and images for: NSUN2 mediated-aberrant 5-methylcytosine methylation regulates autophagy-related ferroptosis in oral squamous cell carcinoma progression
Source: Cell Death Dis. 2025 Dec 23;16(1):903. doi: 10.1038/s41419-025-08174-y (PMC12728175; doi:10.1038/s41419-025-08174-y)

Fig.1K

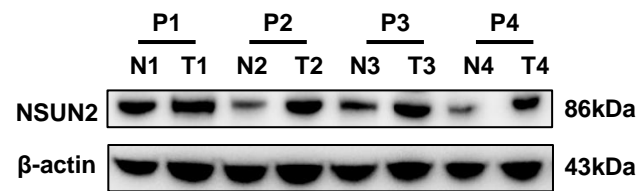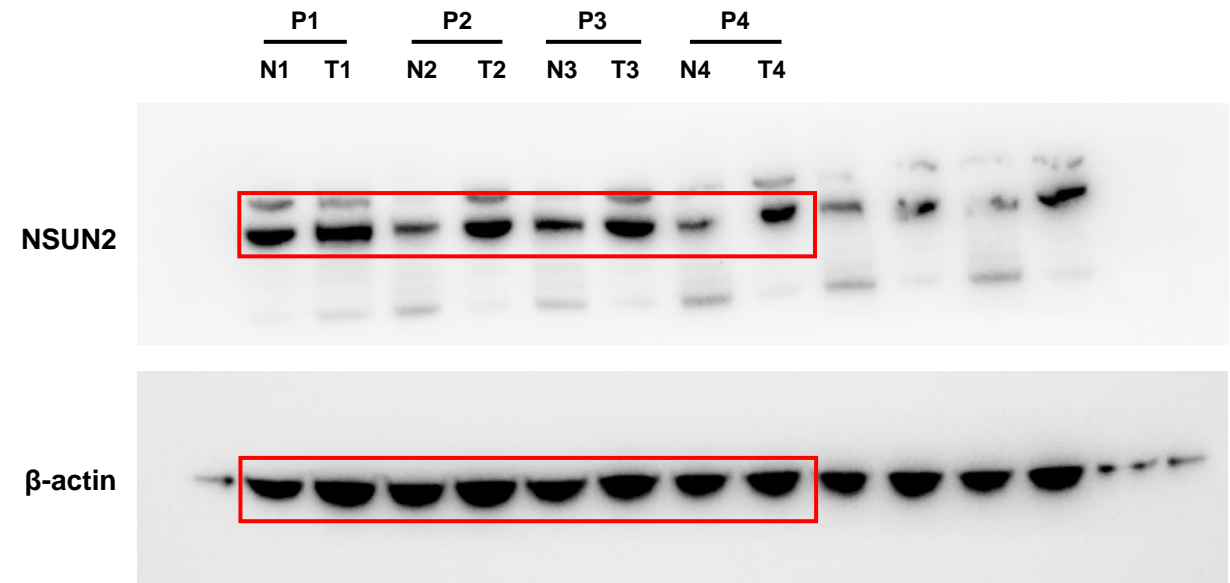

Fig.1I

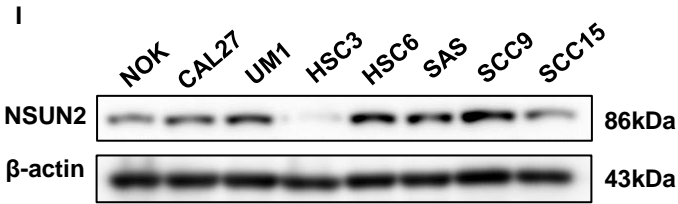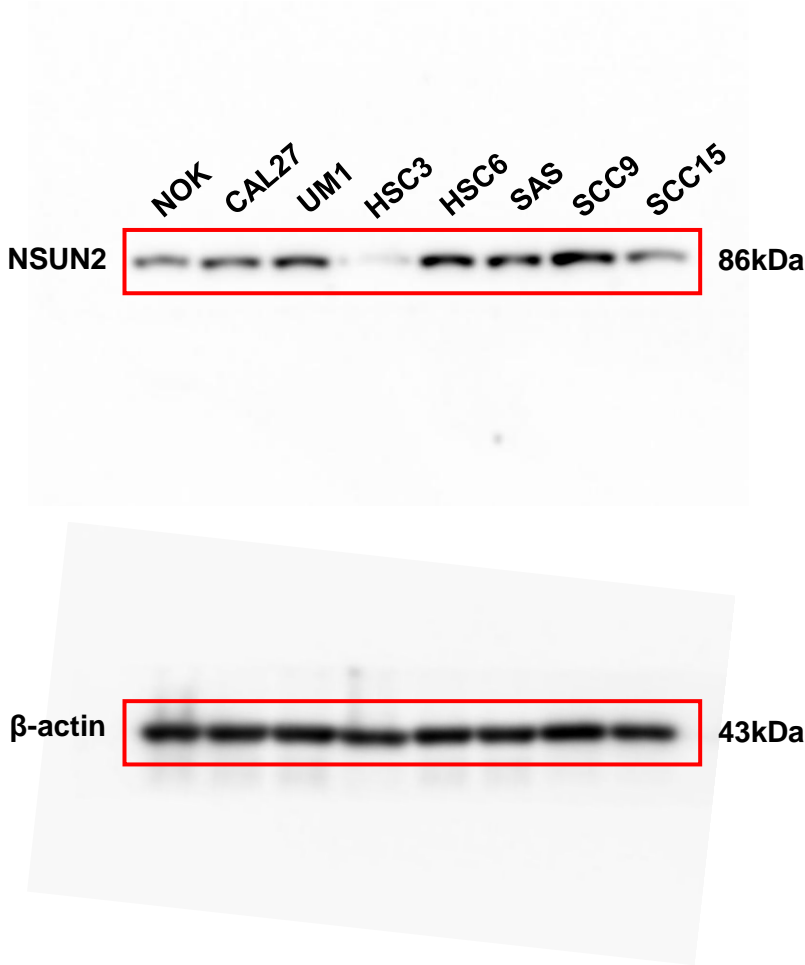

Fig.2B

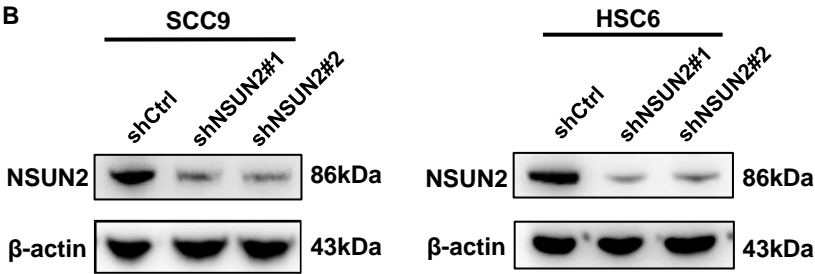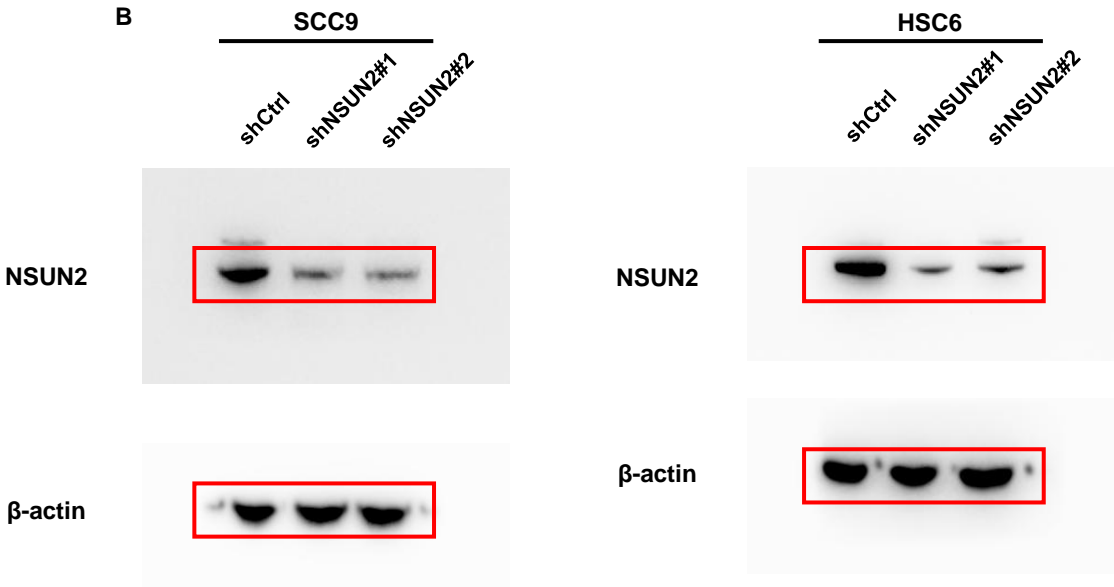

Fig.3B

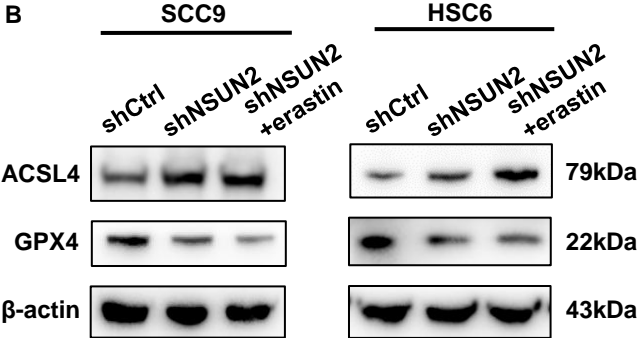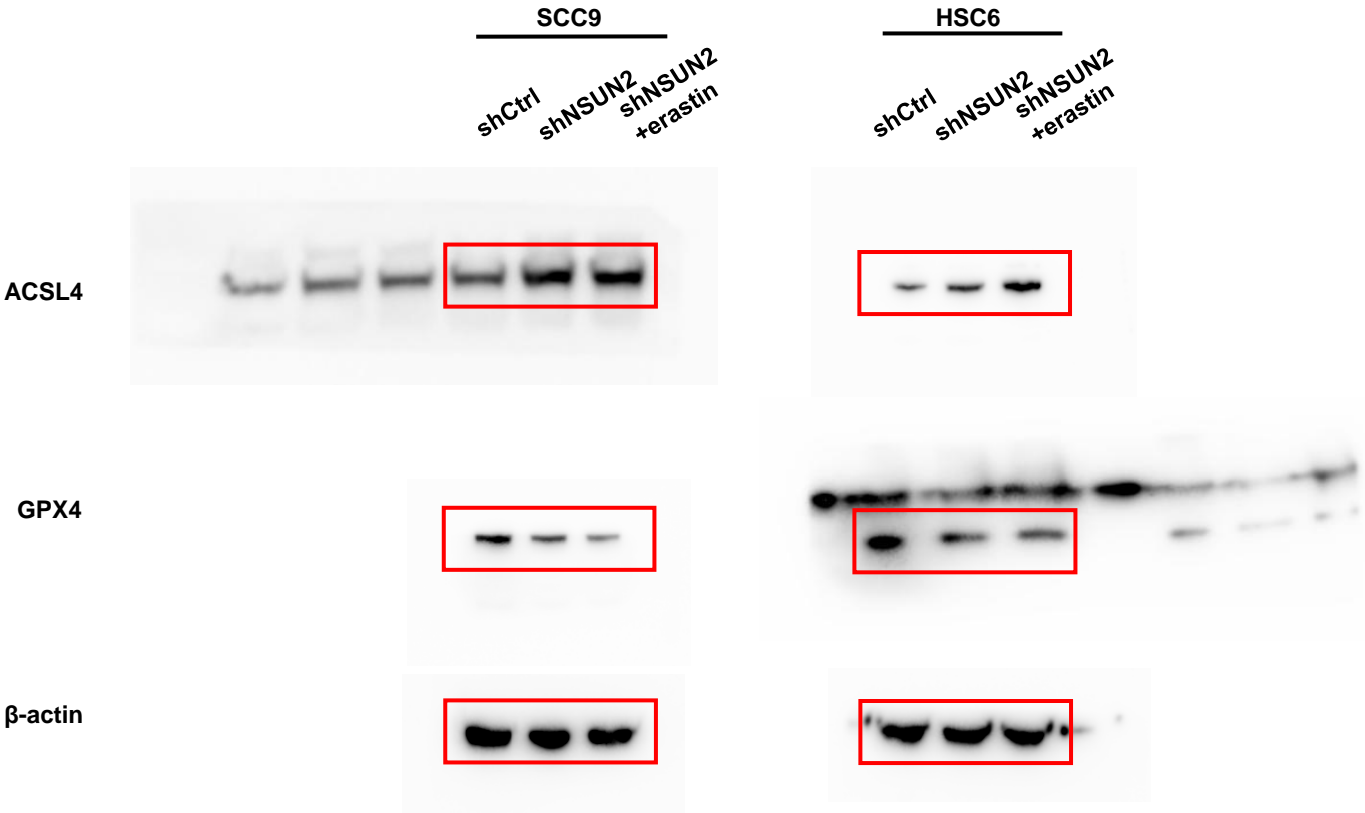

Fig.3G

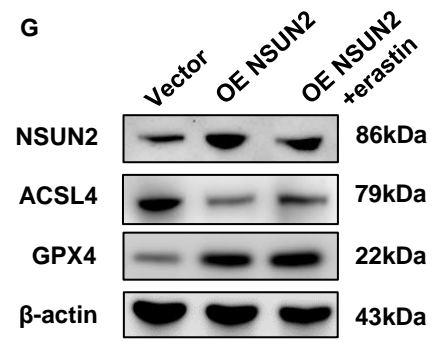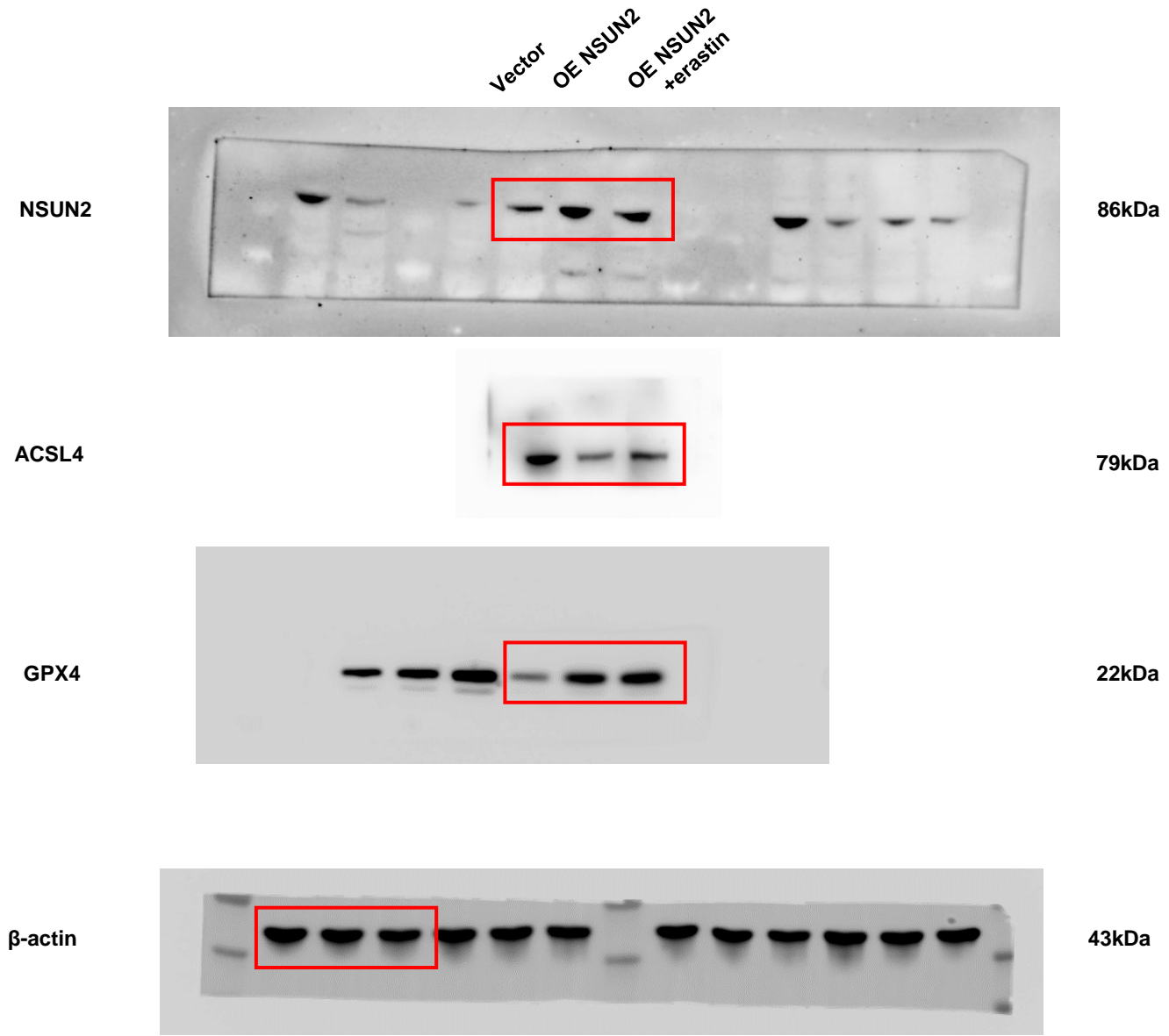

Fig.5A

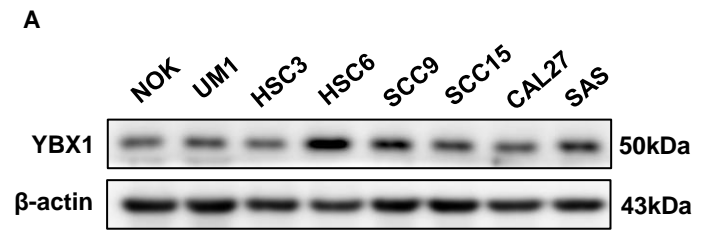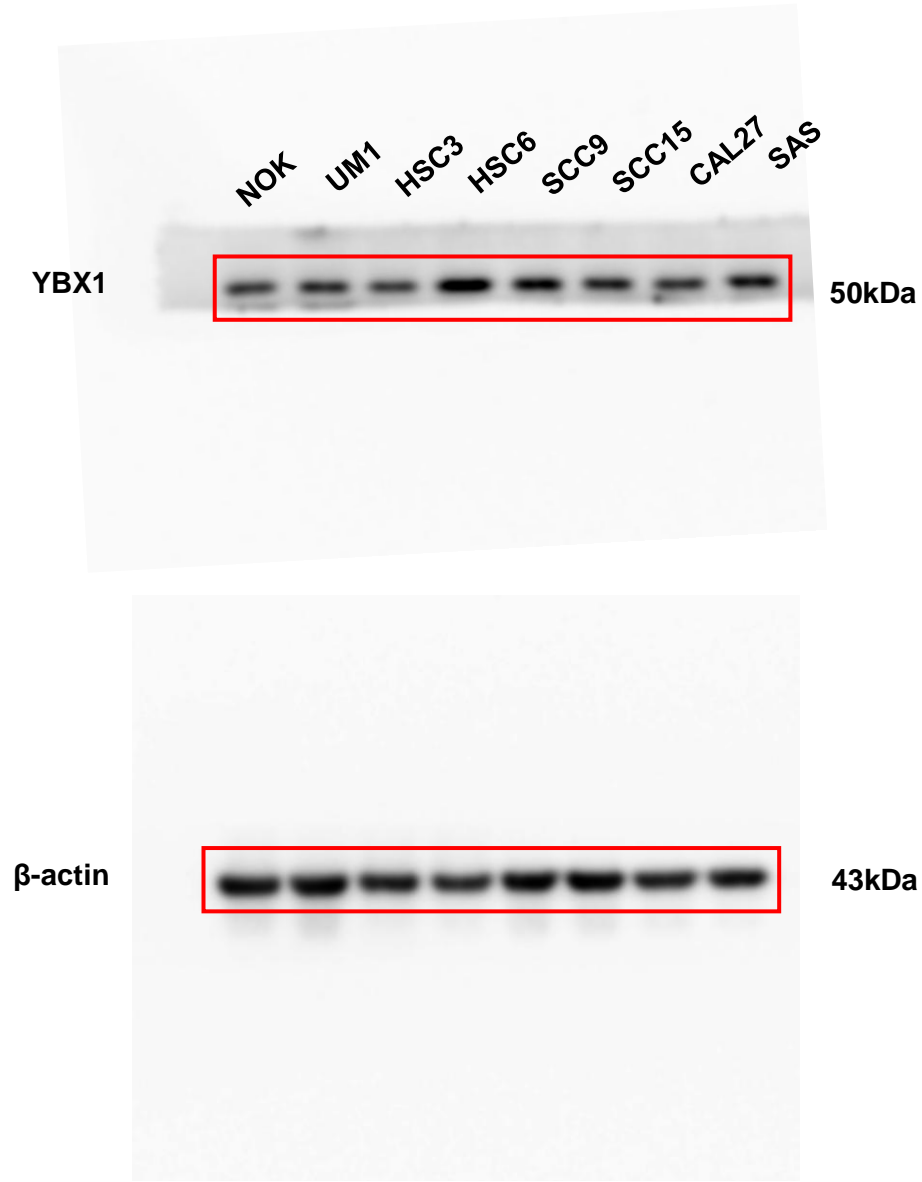

Fig.5D

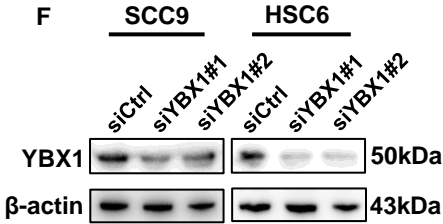

YBX1

β-actin

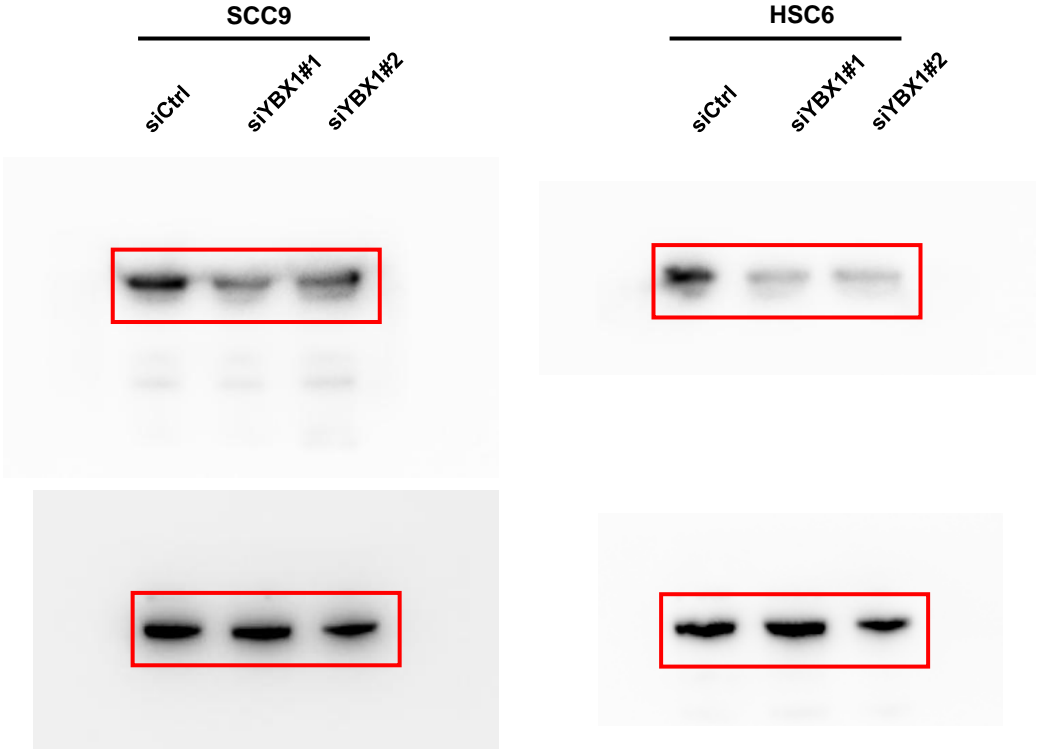

Fig.6A

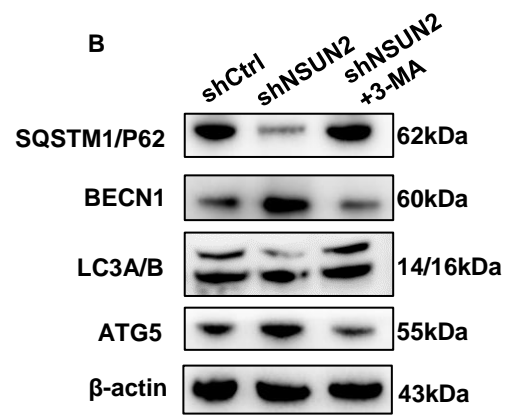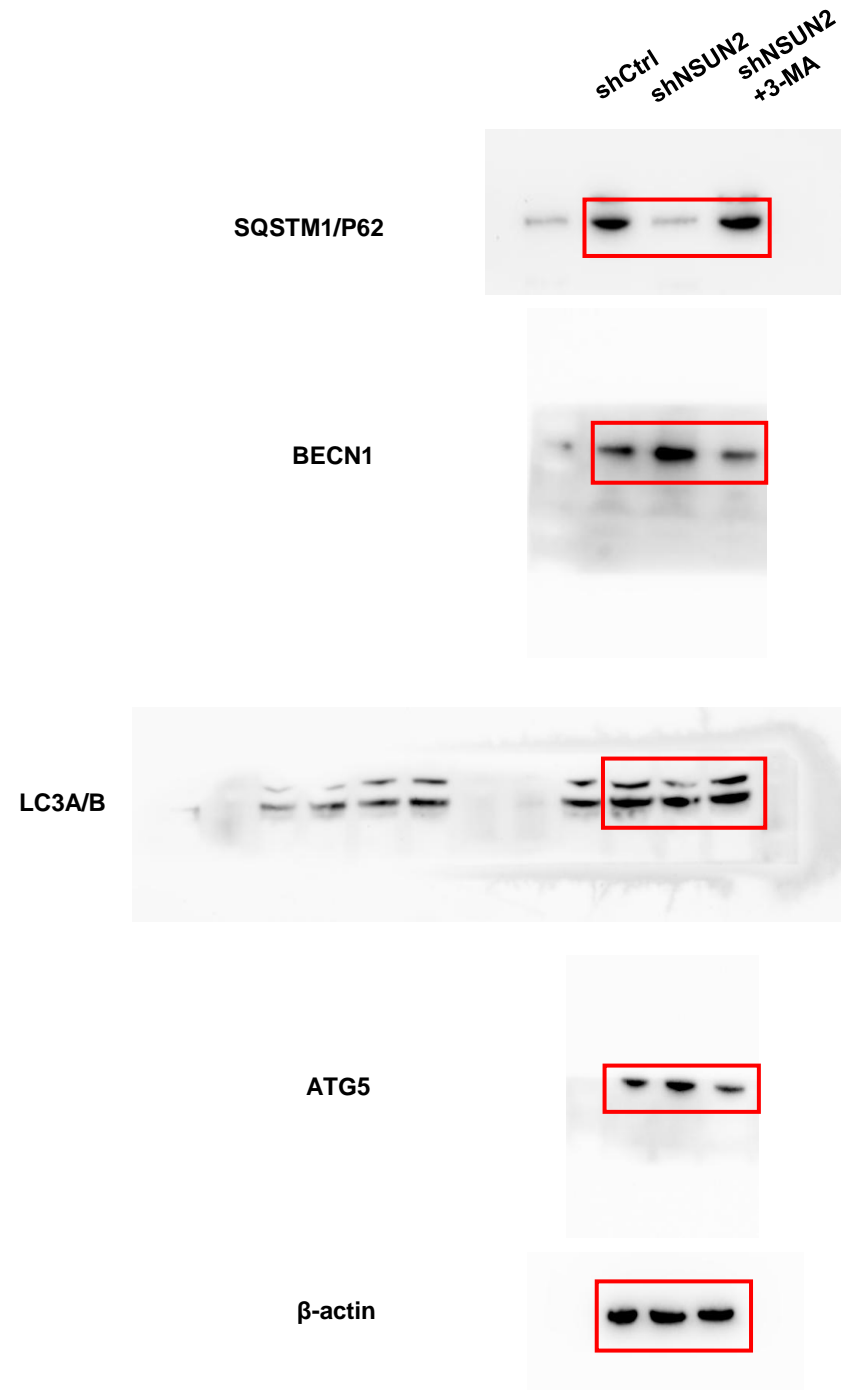

Fig.S2A

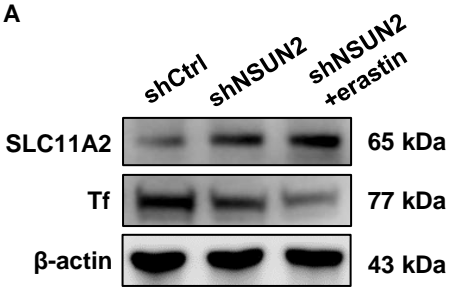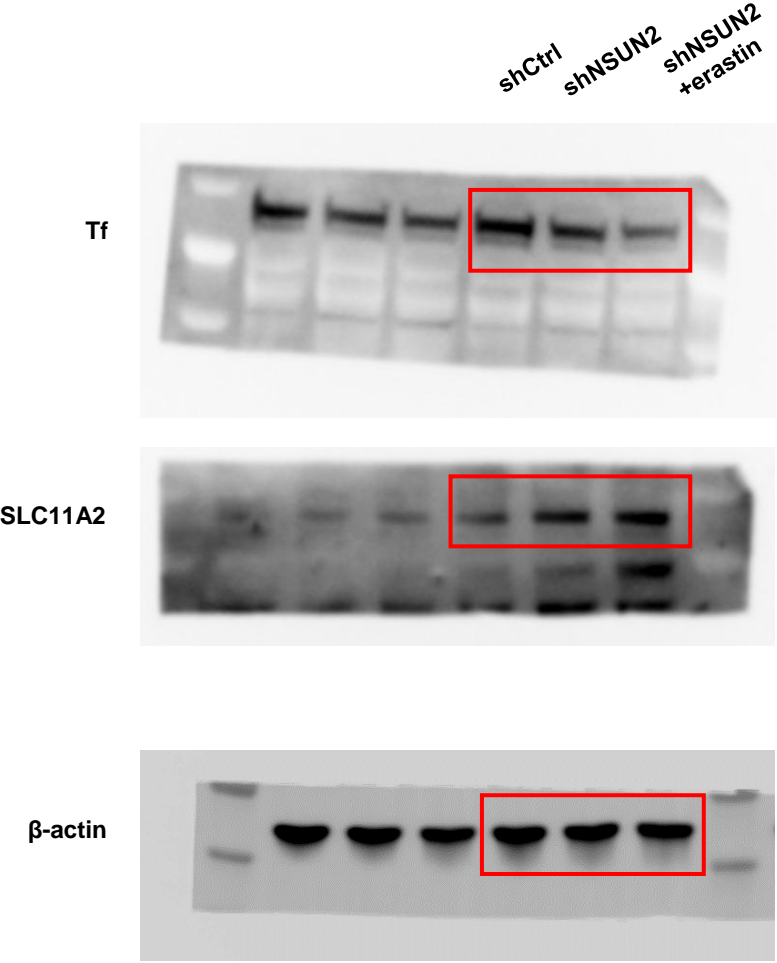

Supplement: Supplementary file 2 — wb original data [file 41419_2025_8174_MOESM2_ESM.pdf]
